# A 3D In-vitro model of the human dentine interface shows long-range osteoinduction from the dentine surface

**DOI:** 10.1038/s41368-024-00298-9

**Published:** 2024-05-11

**Authors:** William Macalester, Asme Boussahel, Rafael O. Moreno-Tortolero, Mark R. Shannon, Nicola West, Darryl Hill, Adam Perriman

**Affiliations:** 1 School of Cellular and Molecular Medicine, Biomedical Sciences Building, University of Bristol, University Walk, Bristol, United Kingdom; 2 Bristol Centre for Functional Nanomaterials, HH Wills Physics Laboratory, University of Bristol, Tyndall Avenue, Bristol, United Kingdom; 3 Centre for Protolife Research, School of Chemistry, University of Bristol, Cantocks Close, Bristol, United Kingdom; 4 Periodontology, Bristol Dental School, University of Bristol, Lower Maudlin Street, Bristol, United Kingdom; 5 Max Planck-Bristol Centre for Minimal Biology, School of Chemistry, University of Bristol, Bristol, United Kingdom

**Keywords:** Molecular medicine, Mesenchymal stem cells

## Abstract

Emerging regenerative cell therapies for alveolar bone loss have begun to explore the use of cell laden hydrogels for minimally invasive surgery to treat small and spatially complex maxilla-oral defects. However, the oral cavity presents a unique and challenging environment for in vivo bone tissue engineering, exhibiting both hard and soft periodontal tissue as well as acting as key biocenosis for many distinct microbial communities that interact with both the external environment and internal body systems, which will impact on cell fate and subsequent treatment efficacy. Herein, we design and bioprint a facile 3D in vitro model of a human dentine interface to probe the effect of the dentine surface on human mesenchymal stem cells (hMSCs) encapsulated in a microporous hydrogel bioink. We demonstrate that the dentine substrate induces osteogenic differentiation of encapsulated hMSCs, and that both dentine and β-tricalcium phosphate substrates stimulate extracellular matrix production and maturation at the gel-media interface, which is distal to the gel-substrate interface. Our findings demonstrate the potential for long-range effects on stem cells by mineralized surfaces during bone tissue engineering and provide a framework for the rapid development of 3D dentine-bone interface models.

## Introduction

Craniomaxillofacial alveolar bone defects arise from a broad range of conditions, including periodontitis, tooth pulpal infection, orthodontic procedures, oral cancer, osteoporosis, and trauma. Traditional clinical therapies for the repair of lost alveolar bone tissue typically involve the implantation of osteoconductive materials.^1,2^ Autologous bone grafts from the mandible for minor bone defects and fibula, rib, iliac crest, and scapula bone grafts for extensive oral reconstruction are the current clinical gold standard, as they have a lower risk of immune rejection, however, they suffer from donor site morbidity, extended surgical times and chronic pain.^1,2^ Bone-like substitute materials, such as hydroxyapatite,^3^ β-tricalcium phosphate (βTCP)^4,5^ and demineralized bone/dentine matrices,^6,7^ have also been investigated, and can be prefabricated into the desired structure for filling the defect *via* bottom-up or top-down approaches. However, the use of materials that require prefabrication are often not conducive to the common small and spatially complex maxilla-oral defects.^8–10^ Accordingly, injectable hydrogel matrices are emerging as promising alternatives to traditional scaffold implants for periodontal tissue engineering and regeneration.^8–11^ Injectable hydrogels that encapsulate stem cells and potentially other bioactive molecules are of key clinical interest, as they can deform upon injection to fill the defect and provide a route to the delivery of high numbers of stem cells to the injury site.^8–11^

Dentine is a highly specialized oral hard tissue with osteoinductive capacity, as demonstrated both in vitro in 2D models and in murine bone fracture models.^12–14^ Whilst synthetic calcium phosphate surfaces have been shown to be osteoinductive to mesenchymal stem cells (MSCs) cultured in 2D monolayers,^15,16^ dentine may further contribute to differentiation from matrix-bound osteoinductive growth factors, which may be liberated by mild demineralization treatments.^7,17^ Moreover, the unique unidirectional array of microtubules in dentine^18^ gives rise to microtopographical features that have also found to be osteoinductive to hMSCs in 2D culture.^19–21^ Hydrogels have been loaded with either the acid-soluble proteins of the dentine matrix^22^ or demineralized dentine matrix particles,^23^ resulting in upregulation of odontogenic markers for encapsulated stem cells. However, these approaches utilized homogenous dispersions of the dentine derivatives within hydrogels, which deviates from the clinical scenario for injectable therapies, where there is a macroscopic dentine-hydrogel interface. This interface creates a spatial environment where heterogenous responses could occur during extracellular matrix (ECM) deposition. Indeed, direct dentine surface-derived induction of cells may first occur at the dentin-gel interface, and the effect subsequently transmitted across the hydrogel *via* paracrine signaling. Little work has been done on modeling the dentine surface, with some organ -on-a-chip models reproducing the dentine microchannel structure in PDMS which oversimplified the dentine-pulp environment.^24^ Some models used dentine disks and dental pulp stem cells to assess the cytotoxicity of oral therapies.^25^ Although some authors have looked at biomaterial-biofilm-dentine interface, no in-vitro model investigating the impact of oral bacteria on regenerative cellular therapies has been developed.^26^

**Fig. 1:**
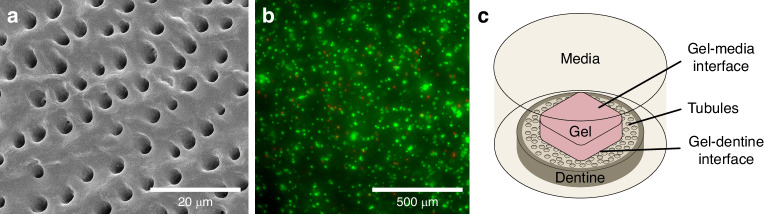
An in vitro model of the dentine-hydrogel interface. **a** A scanning electron microscopy (SEM) image of the human dentine substrate, which contains an array of microtubules at the surface. **b** A widefield fluorescence microscopy image with live (green) dead (red) stains of the hMSC-laden bioink after 3D bioprinting directly onto the dentine substrate. **c** Schematic showing the bioprinted structure with the gel-dentine and gel-media interfacial regions

Accordingly, it is hypothesized that the presence of the dentine surface may affect hMSCs encapsulated within hydrogel across a range of distances, which would impact on the efficacy of injectable stem cell therapies. Here, direct hMSC-dentine interactions would be limited to the gel-dentine interfacial region, but paracrine signaling may yield anisotropic, gradient-based responses in cellular activity across the whole gel. In this work, a hMSC-laden hydrogel-human dentine interface model is developed using extrusion 3D bioprinting (Fig. 1). Using this model, the dentine surface was found to both generate changes in cell morphology and to induce osteogenic differentiation of hMSCs within the hydrogel. Interestingly, extracellular matrix (ECM) production and tissue maturation were found to be more prevalent at the interface distal to the gel-dentine interface, demonstrating long-range effects arising from the dentine surface. Finally, a method for incorporating a representative oral bacterium (*Fusobacterium periodonticum*) within in a 3D bioprinted coculture model with hMSCs is demonstrated, finding that the obligate anaerobic species remains viable after seven days in aerobic culture. Conversely, a significant decrease in hMSC viability was observed after 7 days, which was accompanied by widespread stress responses, such as uniaxial elongation, and the formation of lipid droplets and blebbing, which suggested that the infection led to hMSC death.

## Results

### Human mesenchymal stem cell distribution and viability in bioprinted constructs

Bioprinting has emerged as one of the premier techniques in the field of tissue engineering, enabling the automated, scalable additive-manufacture of 3D constructs with spatio-temporal control.^27–29^ Here, hMSC-laden bioinks were bioprinted directly onto either tissue culture plastic or dentine (Fig. 1c) in a manner analogous to injection to model the hydrogel-dentine interface. Additionally, β-tricalcium phosphate (βTCP) disks were used as a control substrate to elucidate the contribution of the calcium phosphate fraction in dentine, as calcium phosphate surfaces have previously been shown to be osteoinductive to MSCs in 2D.^15,16^ To remove the smear layer occluding the dentine tubules (Supplementary Fig. S1), dentine slices were prepared by exfoliation and sonication with mild acid solution (Fig. 1a).

**Fig. 2:**
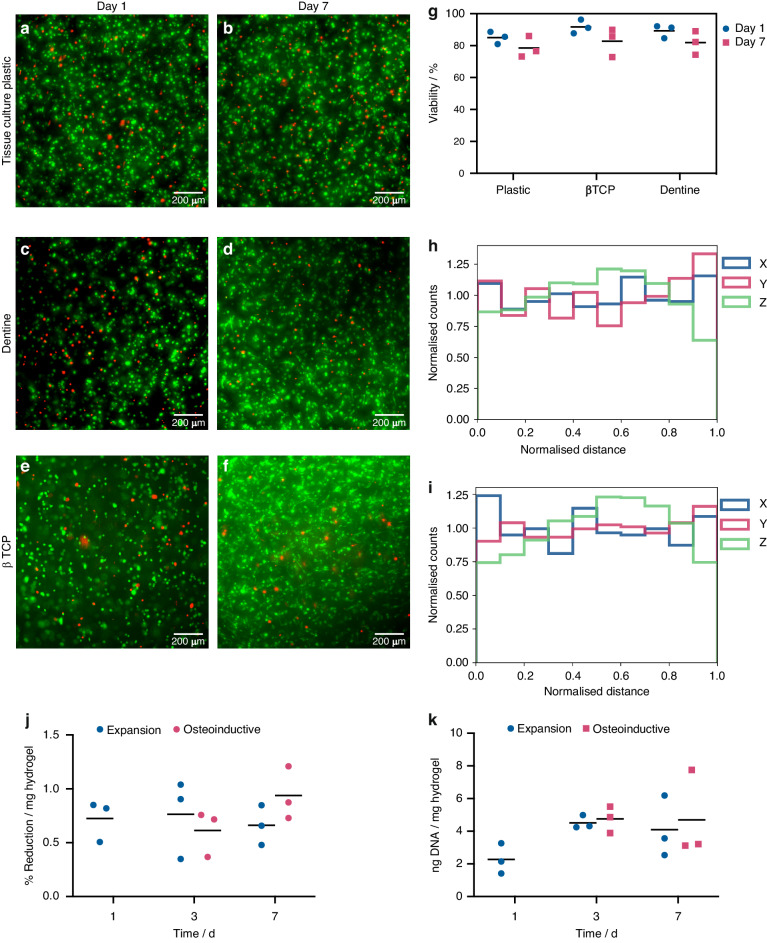
**a**–**f** Cell viability and proliferation of hMSCs on bioprinted constructs. Viability was assessed for constructs bioprinted onto either tissue culture plastic, βTCP disks or dentine slices after 1 or 7 days of culture. **a**–**f** Widefield micrographs of live (Calcein AM, green)/dead (ethidium homodimer-1, red) staining of the bulk of the constructs printed onto culture plastic (**a**, **b**), dentine (**c**, **d**) or βTCP disks (**e**, **f**) after 1 (**a**, **c**, **e**) or 7 (**b**, **d**, **f**) days of culture. hMSCs were predominantly spherical at day 1, with some evidence of polarization at day 7. Analysis was performed in collaboration with Emily Cornwell. **g**–**i**: **g** Cell viability of hMSCs constructs bioprinted onto either tissue culture plastic, βTCP disks or dentine slices after 1 or 7 days of culture. Live/Dead staining was performed using a commercially available kit containing Calcein AM and ethidium homodimer and bars represent the mean. No statistical significance was determined by 2-Way ANOVA (*N* = 3). **h**, **i** Distribution of live hMSCs along the x, y and z axes within bioprinted constructs on tissue culture plastic remained approximately uniform after 1 (**h**) or 7 (**i**) days. **j**, **k** Assessment of the proliferation of hMSCs in bioprinted samples. **j** metabolic activity of hMSCs in bioprinted samples. Samples were cultured in expansion media for 1 day, then split into groups cultured in either expansion media (blue) or osteoinductive media (red) for the rest of the time points. Little change was observed in metabolic activity over 7 days of culture. **k** DNA assays showed no increase over the 7 days of culture. No statistical significance was determined by 2-way ANOVA

The bioink formulation used in this work was a composite of alginate and Pluronic-F127, which was developed in Armstrong et al.^27^ Here, 5.0 × 5.0 × 0.41 mm structures were bioprinted onto 37 °C substrates with 100% infill density using a rectilinear style pattern, where the sol-gel transition of the Pluronic phase provided short-term structural fidelity. This was followed by ionic crosslinking of the alginate phase with a room temperature calcium chloride solution to provide long term structural fidelity, whilst simultaneously driving a gel-sol transition and subsequent removal of the Pluronic porogen. The resulting bioprinted constructs were charachterised to assess their suitability for bone tissue engineering. Bovine serum albumin-conjugated tetramethylrhodamine (BSA-TAMRA) was used to compare the diffusion coefficient (D_eff_) of the bioink with a hydrogel comprising the same mass fraction of alginate (Supplementary Fig. S2). Here, the microporosity of the bioink resulted in a two-fold increase in D_eff_. The compressive Young’s modulus of the printed construct under tissue culture conditions (15 ± 2 kPa, Supplementary Fig. S3) was also found to be within the appropriate range for bone tissue engineering.^30^ The suitability of the bioink formulation, the bioprinting process and the preparation of the mineral substrates were confirmed by high hMSC viabilities (>75%) observed over 7 days of culture (Figs. 1b and 2a–g). The cells were found to be homogenously distributed in the bulk of the construct after 1 or 7 days of culture (Fig. 2h, i, Supplementary Fig. S4) while their proliferative capacity was found to be negligible (Fig. 2j, k).

### The effect of the substrate identity on cell morphology in 3D bioprinted constructs

**Fig. 3:**
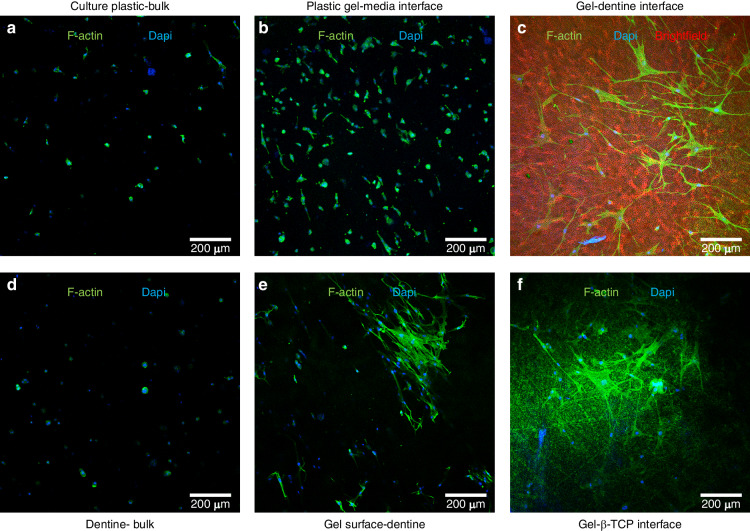
Cell morphology on bioprinted constructs. Immunofluorescence staining for F-actin (green) and DAPI (blue) of cell laden constructs after 7 days culture. hMSCs were predominantly spherical within the bulk of the constructs on culture plastic and dentine (**a**, **d**). Small actin protrusions were observed at the gel-media interface for constructs on culture plastic (**b**), whereas widespread polarization of hMSCs was observed at the gel-dentine interface (**c**). the red shows the dentine tubules in brightfield. **e** After observation of cell polarization at the gel-dentine interface (**c**), the gel was carefully removed and highly polarized cells were found anchored at the corresponding gel surface. **f** Cells were observed to polarize at the gel-βTCP interface for constructs cultured on βTCP disks

The morphology of the bioprinted hMSCs was investigated after 7 days culture by f-actin immunofluorescence (Fig. 3). hMSCs were predominantly spherical for all samples within the bulk gel (Fig. 3a, d). However, cell polarization was evident at the gel-media interface (Fig. 3b) and the gel-dentine interface (Fig. 3c). To confirm that these highly polarized cells remained anchored within the bioink, the constructs were removed from the dentine substrates and the corresponding surface of the hydrogel imaged, finding highly polarized cells at the surface (Fig. 3e). Cell polarization was also observed at the gel-βTCP interface (Fig. 3f).

### Spatial dependency of surface-driven osteoinduction

**Fig. 4:**
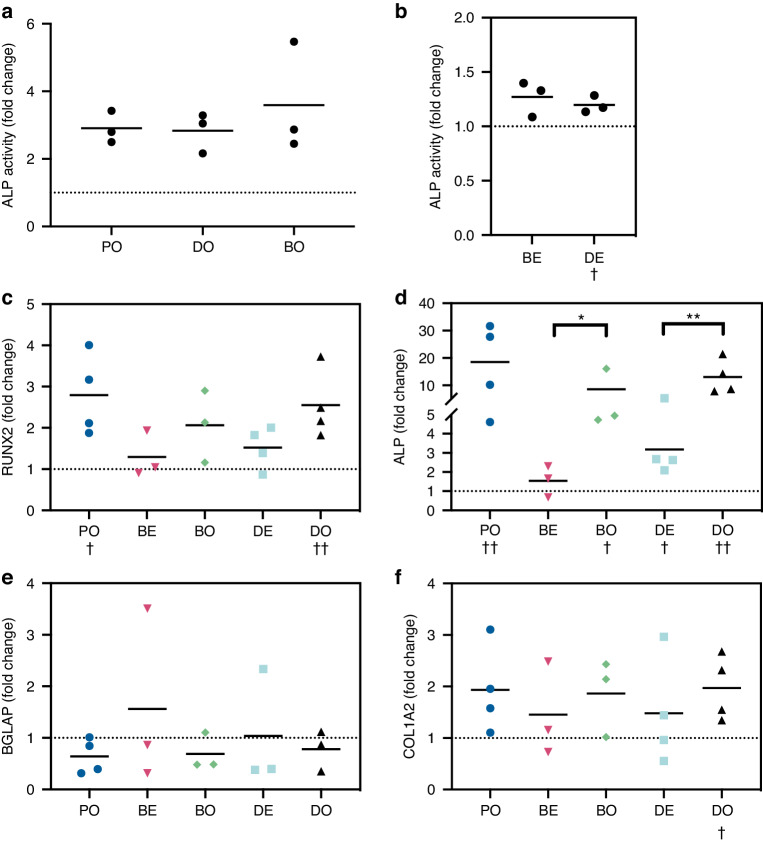
Analysis of hMSC differentiation within constructs bioprinted onto either tissue culture plastic, dentine or β-TCP disks. **a** ALP activity assays for samples cultured on tissue culture plastic, dentine or β-TCP compared in osteoinductive media for 7 days to culture plastic in expansion media (dotted line). **b** ALP activity assays after 7 days of culture in expansion media shows upregulation for samples on dentine compared to culture plastic (dotted line). ALP activity was first normalized to mass of hydrogel per sample, and statistical significance determined by RM one-way ANOVA (†*P* < 0.05, *N* = 3). The data were then converted to fold change relative to PE for plotting. Bars represent the mean. **c**–**f** Gene expression analysis of constructs bioprinted onto either tissue culture plastic (P, *N* = 4), dentine (D, *N* = 4) or β-TCP disks (B, *N* = 3) and cultured either in expansion (E) or osteo media (O) for 14 days. PE (culture plastic in expansion media), PO (culture plastic in osteoinduction media), DE (Dentine slices in expansion media), DO (Dentine slices in osteoinduction media), BE (β-TCP disks in expansion media), BO (β-TCP disks in osteoinduction media). The results were normalized against PE (dotted line), and the data presented fold changes. Gene expression analysis for RUNX2 (**c**) revealed that hMSCs may be induced to undergo osteogenic differentiation using an osteoinductive media regardless of the substrate (PO, DO, BO), as well as a trending upregulation for samples in expansion media on dentine (DE). These trends were conserved for gene expression analysis of ALP (**d**), which demonstrated a statistically significant upregulation. BGLAP (**e**) expression trended towards downregulation indicating that cells have not reached the mature osteoblast stage. COL1A2 (**f**) trended towards upregulation with significance for DO indicting immature osteoblasts. All statistical analysis was performed on ΔΔCT values. Paired t-tests (ΔCT plastic expansion vs ΔCT treatment, †*P* < 0.05, ††*P* < 0.01) were used to investigate autoinduction of the gene compared to PE. 2-way ANOVA on ΔΔCT values was used to compare statistical differences between groups (*P < 0.05, **P < 0.01, ***P < 0.001, ****P < 0.000 1). Bars represent the mean

To assess hMSC differentiation within the bioprinted constructs, gene expression analysis was performed at day 14 (Fig. 4c, d). Runt-related transcription factor 2 (RUNX2) is a master regulator for osteogenesis and is one of the furthest upstream transcription factors associated with osteoblast differentiation.^31^ Alkaline phosphatase (ALP) is an enzyme that is expressed early in the mineralization process, with levels of expression linked to the degree of downstream mineralization.^32,33^ Upregulation of RUNX2 and ALP were observed for all samples cultured with osteoinductive media. Interestingly, the dentine substrate also yielded significant upregulation of ALP and a trending upregulation in RUNX2 in expansion media. This finding was corroborated by a significant increase in ALP enzymatic activity at day 7 (Fig. 4a, b). Nonetheless the differentiation seems to generate immature osteoblasts as indicated by the downregulation of BGLAP and the upregulation of COL1A2 (Fig. 4e, f).

**Fig. 5:**
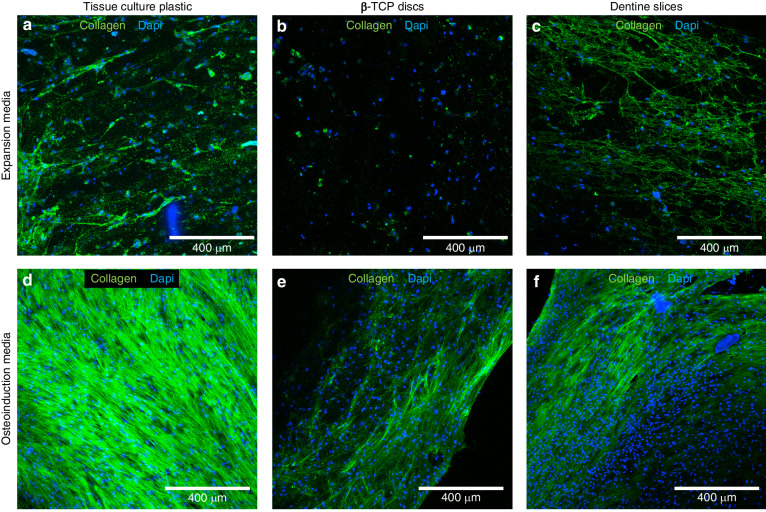
Collagen deposition for hMSCs constructs bioprinted onto either tissue culture plastic, βTCP disks or dentine slices. Representative max projections of immunofluorescence micrographs for collagen I (green) counterstained for cell nuclei (blue, DAPI). Samples (*N* = 3) were bioprinted onto either tissue culture plastic (**a**, **d**), βTCP disks (**b**, **e**) or dentine slices (**c**, **f**) and cultured with either expansion (**a**–**c**) or osteoinductive media (**d**–**f**) for 28 days. Samples cultured with osteoinductive media all showed a thin fibrous mat at the gel-media interface accompanied with a high density of cell nuclei, with minimal staining within the bulk. With samples cultured on tissue culture plastic (**d**) showing the highest collagen deposition. Samples cultured with expansion media were imaged at greater laser powers, with widespread positive staining observed in the cytoplasm of hMSCs in the bulk of constructs cultured on dentine slices

To assess the spatial dependency of surface-driven osteoinduction, hMSCs within bioprinted constructs on either tissue culture plastic (Fig. 5a, d), β-TCP disks (Fig. 5b, e) or dentine slices (Fig. 5c, f) were cultured with either expansion (Fig. 5a–c) or osteoinductive media (Fig. 5d–f), with immunofluorescent staining of collagen type 1 performed after 28 days of culture (Fig. 5). Collagen type 1 is the most abundant protein in bone and is therefore a key marker for bone tissue engineering.^34^ Samples cultured in osteoinductive media all exhibited a dense layer of co-aligned collagen type 1 fibers at the gel-media interface accompanied by high cell populations, with samples cultured on tissue culture plastic (Fig. 5d) exhibiting the highest collagen deposition. Samples cultured with expansion media did not possess a layer at the gel-media interface, except for the observation of extensive cytoplasmic staining at greater laser powers for samples cultured on dentine (Fig. 5c) compared to the other substrates. This was corroborated with the increased opacity for bioprinted constructs cultured in osteoinductive media after 4 weeks (Supplementary Fig. S5). This is most likely indicative of ECM formation and mineral deposition, as would be expected from the increase in ALP activity and upregulation of osteogenic genes at earlier time points. Interestingly, an increase in the opacity of samples cultured on dentine compared to tissue plastic was also observed when removed from the dentine slice. This observation lends credence to the autoinductive properties of the substrate as observed by ALP activity and gene expression analysis. These autoinductive properties of the dentine substrate were further corroborated by nanoindentation analysis (Supplementary Fig. S6) of the bioprinted constructs after 5 weeks of culture which revealed that the Young’s modulus at the gel surface greatly increased when cultured with osteoinductive media for 5 weeks. The modulus distribution was observed to shift to a higher value range for samples cultured on dentine, with a higher shift found at the gel-dentine interface compared to the gel-media interface. These results further corroborate the autoinductive properties of the dentine substrate.

**Fig. 6:**
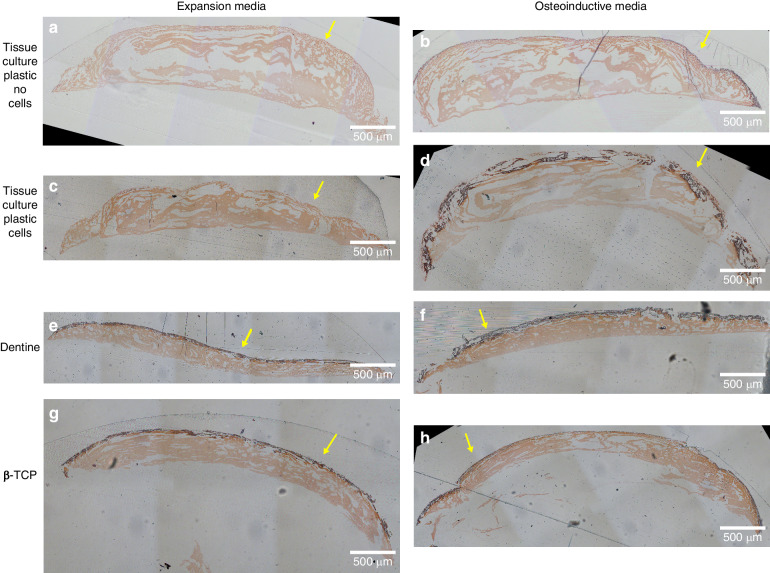
Glycosaminoglycans deposition for hMSCs constructs bioprinted onto either tissue culture plastic, βTCP disks or dentine slices Representative histological images of safranin O staining of constructs bioprinted (*N* = 3) with (**c**–**h**) or without (**a**, **b**) hMSCs, onto either tissue culture plastic (**a**–**d**), dentine slices (**e**, **f**) or βTCP disks (**g**, **h**) and cultured for 28 days in expansion media (**a**, **c**, **e**, **g**) or osteoninductive media (**b**, **d**, **f**, **h**). Samples were removed from the substrates and embedded in LRW and sectioned (1 mm thick). Minimal positive staining (black) was observed for the acellular controls and for cellular samples cultured in expansion media. Alginate background staining (light orange) was observed in all samples, and glycosaminoglycan staining (dark red/black) was observed at the gel-media interface (yellow arrows) for either substrate. A thick band of positive staining was observed at the gel-media interface (yellow arrows) for cellular samples cultured with osteoinductive media

**Fig. 7:**
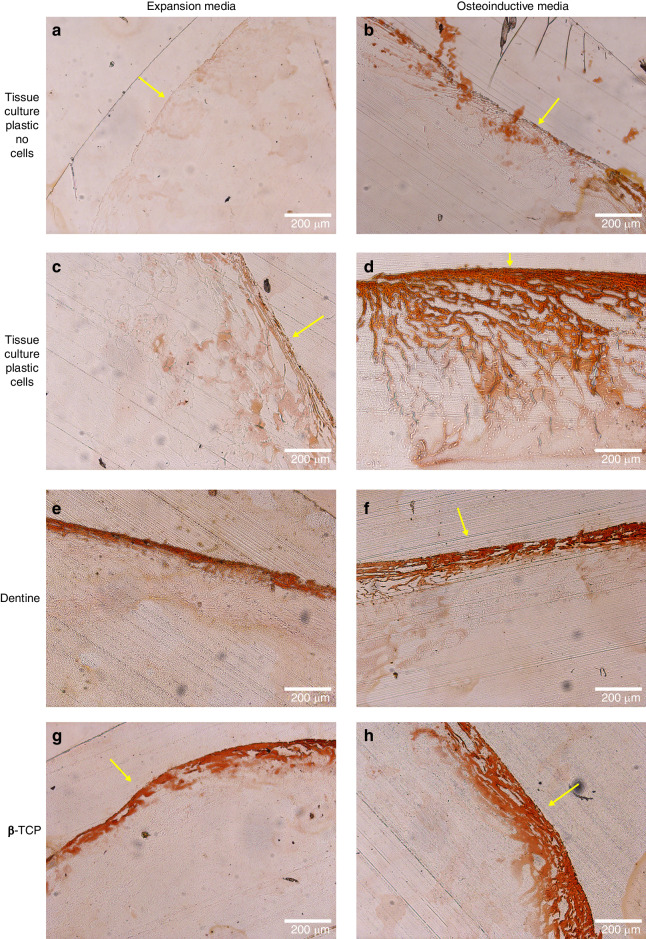
Mineralization for hMSCs constructs bioprinted onto either tissue culture plastic, βTCP disks or dentine slices Representative histological images of alizarin red staining of constructs bioprinted (*N* = 3) with (**c**–**h**) or without (**a**, **b**) hMSCs onto either tissue culture plastic (**a**–**d**) dentine slices (**e**, **f**) or βTCP disks (**g**, **h**) and cultured for 28 days in expansion media (**a**, **c**, **e**, **g**) or osteoinductive media (**b**, **d**, **f**, **h**). Minimal positive staining was observed for the acellular controls and for cellular samples cultured in expansion media. However, a thick band of positive staining was observed at the gel-media interface (yellow arrows) for cellular samples cultured with osteoinductive media and at the gel-media interface for either substrate (yellow arrows)

To confirm the distribution of engineered ECM within the bioprinted constructs, samples were cultured for 28 days prior to embedding in London Resin white (LRW), sectioning and staining. Histological staining for glycosaminoglycans (safranin O, Fig. 6) and calcium deposits (alizarin red, Fig. 7) both yielded positive staining at the gel-media interface for samples cultured on dentine and βTCP in expansion media, but not for samples cultured on tissue culture plastic. These staining profiles were similarly observed for samples grown in osteoinductive media.

The formation of hydroxyapatite mineral deposits is commonly used to assess the maturation of engineered bone tissue. Significantly, the mineral deposits within the bioink were found to be biologically derived hydroxyapatite by FTIR (Supplementary Fig. S7), PXRD (Supplementary Fig. S8) and SEM/EDX (Supplementary Fig. S9). The mineral distribution across the whole construct, as opposed to individual sections, was confirmed by μCT (Supplementary Fig. S10), finding regions of high electron density primarily located at the gel-media interfacial regions.

**Fig. 8:**
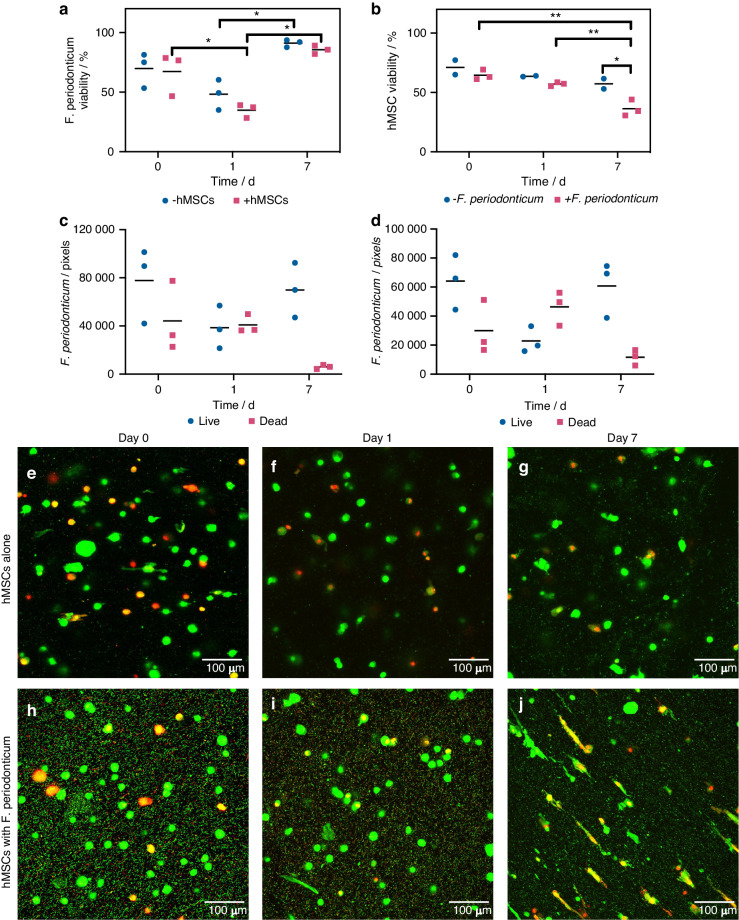
Investigating coculture of hMSCs and Fusobacterium periodonticum. **a** Viabilities of F. periodonticum over 7 days of 3D culture in expansion media (*N* = 3). There was a decreasing trend in viability after 1 day of culture, and a resurgence in viability by day 7 in monoculture (blue). The introduction of hMSCs (pink) did not yield a significant deviation from this profile. **b** Viabilities of hMSCs over 7 days of 3D culture in expansion media (*N* = 3). Coculture with F. periodonticum (pink) yielded a significant decrease in hMSC viability at day 7 compared to monoculture (blue). Statistical significance was determined via Two-way ANOVA (*P < 0.05, **P < 0.01). Bars represent the mean. Pixel counts used for F. periodonticum viability analysis in either monoculture (**c**) or coculture with hMSCs (**d**). Bars represent the mean. A large decrease in the number of dead pixels was observed for both sample types between days 1 and 7, accompanied by an increase in live pixels. **e**–**j** Representative confocal micrographs of total nucleic acid (green, Syto9)/dead (red, propidium iodide) staining of hMSCs after culture. At days 0 and 1 of culture, hMSCs exhibited a predominantly spherical morphology in both monoculture (**e**, **f**) and coculture (**h**, **i**). After 7 days of culture (**g**, **j**), hMSCs exhibited a predominantly spherical morphology in monoculture (**g**) but exhibited uniaxial elongation in coculture (**j**) with evidence of lipid droplet formation

Due to the proximity of the oral microbiome, models for oral healthcare therapies should investigate the impact of representative oral bacteria due to the potential for infection. Previous studies investigating 3D eukaryote-prokaryote coculture have focused on building models of the gingival tissues and the oral mucosa rather than injectable hydrogel therapies.^35,36^ In this study, the potential for 3D bioprinted in vitro models of eukaryote-prokaryote interactions was further explored by the creation of a coculture of *Fusobacterium periodonticum* and hMSCs in the bioink. *F. periodonticum* are obligately anaerobic, non-motile, gram-negative rod bacteria that are a member of the *Fusobacterium* genus,^37^ which are opportunistic pathogens that are a key bridging species in the formation of plaque.^38,39^ Here, viable *F. periodonticum* were incorporated into the bioink along with hMSCs for homogenous seeding (Supplementary Fig. S11), representing a widespread infection of the injectable therapy. The viability of *F. periodonticum* in 3D monoculture was first determined over 7 days (Fig. 8a), finding a general decrease after 1 day followed by a resurgence after 7 days. The total population was not found to greatly increase between days 1 and 7 by comparison of total pixel counts from live/dead microscopy images (Fig. 8c), and the presence of gram-negative rods in the constructs after 7 days was confirmed by gram staining (Supplementary Fig. S12).

A similar profile for the viability of *F. periodonticum* was observed upon addition of hMSCs in coculture (Fig. 8a, d). However, a significant decrease in hMSC viability was observed after 7 days in coculture compared to monoculture (Fig. 8b). Moreover, hMSCs in coculture (Fig. 8j) were found to exhibit widespread stress responses, such as uniaxial elongation, the formation of lipid droplets and blebbing,^40–42^ when compared to the spherical morphology in 3D monoculture (Fig. 8g) and in coculture at earlier timepoints (Fig. 8h, i).

## Discussion

The developed bioink formulation and design were shown to have appropriate properties for the proposed application. This includes mechanical properties in a suitable range for bone tissue engineering applications and sufficient microporosity to support improved mass transport properties of the gel. The bioink supported the growth and differentiation of hMSCs with a viability of up to 75% over 7 days. The small decrease in viability observed across all conditions between day 1 and day 7 was attributed to the persistence of dead cells trapped in the construct during media changes (Fig. 2g), especially with the limited proliferation of hMSCs in the bioprinted constructs as determined using metabolic activity and DNA assays (Fig. 2j, k). The even distribution of the cells within the bioprinted constructs (Fig. 2h, i) suggests that issues arising from large bioprinted constructs, such as central zone necrosis and cell sedimentation were not significant.^43^ Their limited proliferation is consistent with previous studies where hMSCs were found to adopt a quiescent state within the bulk alginate hydrogels.^44,45^ Thus demonstrating the suitability of the model for investigating the spatial dependence of ECM deposition and tissue maturation within the hydrogel, as the cells remained viable and homogenously dispersed.

hMSCs encapsulated within the bulk of the gel maintained a spherical morphology (Fig. 3a, d) due to the lack of binding sites for cellular mechanotransduction in the alginate network. However, the observed high cell polarization at the gel media interface (Fig. 3b), the gel-βTCP interface (Fig. 3f), and at the gel-dentine interface (Fig. 3c, e) confirms the ability of these cells to interact and respond to growth factors in the media and surrounding surfaces. At the dentine surface, the cells were also shown to remain anchored and polarized when the gel-dentine interfacial region was imaged.

The bioactivity of the dentine surface has been previously demonstrated, with cellular processes in new bone tissue found to invade the dentine tubules,^6,46^ and genes associated with odontogenesis were found to be upregulated for cells in close proximity to the surface.^46^ Due to the observed interaction between hMSCs and the substrates, it was therefore hypothesized that the substrates could be osteoinductive to hMSCs in the 3D constructs. This was confirmed with gene expression analysis. Elevated gene expression of ALP and RUNX2 in hMSCs differentiated in osteoinductive media within the bioprinted constructs for 14 days confirms the compatibility of the bioink formulation and the substrates with hMSC differentiation. Upregulation was also noted with the dentine substrate in expansion media suggesting that the substrate was autoinductive to hMSCs cultured in 3D over 14 days. This, along with the increased ALP enzymatic activity at day 7, confirms the hypothesis that the dentine substrate would be osteoinductive towards hMSCs in the bioprinted constructs. However, it should be noted that the results from these assays represent an average of the entire hMSC population for a given sample. The 3D spatial dependence of the induction may not therefore be inferred – this may be limited to the layers of cells near the gel-dentine interface, or there may be induction occurring across the whole construct.

Collagen fiber deposition was assessed to further confirm the differentiation of hMSCs within the bioprinted constructs. In samples cultured with osteoinductive media, collagen fibers were only deposited at the gel-media interface (Fig. 5d–f), which may result from diffusion-limited transport of growth factors into the bioprinted constructs, or from hMSCs in the bulk entering a quiescent-like state. There was no collagen deposition in the bulk or at the gel-substrate interface (data not reported). For these samples cultured in osteoinductive media, collagen deposition seemed to be higher in samples cultured on tissue culture plastic (Fig. 5d). In this instance, collagen deposition is driven by the osteoinductive media, and the ease of penetration of imaging and staining reagents could explain the higher collagen staining for samples cultured on tissue culture plastic compared to β-TCP and dentine disks For samples cultured with expansion media, the absence of fibers may be due to the omission of ascorbic acid; an essential amino acid for collagen crosslinking to form fibrous structures in the media.^47^ Samples cultured on dentine however, showed more cytoplasmic staining. It may therefore be hypothesized that the dentine surface stimulates the formation of collagenous structures reminiscent of the periodontal ligaments, an important connective tissue in the periodontium comprised primarily of collagen type 1 and hydroxyapatite.^48^ The regeneration of periodontal ligament structures would be significant, providing better anchorage of the implantable material into the wound site and should be investigated further.

The deposition of ECM was further confirmed by the histological staining for glycosaminoglycans and calcium deposits, which similarly was concentrated at the gel-media interface in cells cultured in expansion media (Figs. 6 and 7). The presence of glycosaminoglycans and calcium deposits in the region distal to the gel-dentine interface demonstrate long-range induction by the surface for ECM production and tissue maturation. Removal of the sample from the dentine disks was required for processing, and it is possible that a highly adherent cell layer may have detached from the gel at the gel-dentine interface and thus would not be observed. In samples grown on osteoinductive media, the similar staining profiles could be partly due to the diffusion-limited transport of growth factors across the hydrogel. FTIR, SEM/EDX and mCT results confirm that the formation of hydroxyapatite mineral deposits associated with bone tissue engineering predominantly occurred at the gel-media interface, distal to the gel-substrate interface.

The results presented here demonstrate the osteoinductive potential of a dentine substrate towards hMSCs encapsulated in an adjacent 3D hydrogel. Encapsulated hMSCs were found to differentiate down osteogenic lineages when bioprinted onto a dentine substrate, and ECM production and tissue maturation were predominantly localized to the gel-media interface, distal to the gel-substrate interface. This spatial complexity may arise from a combination of several different factors. The absence of ECM production in the bulk may have arisen due to limited diffusion of nutrients from the media resulting in a critical zonal depth, or hMSCs entering a quiescent-like state. Moreover, hMSCs in direct contact with the substrate surface may have begun to secrete paracrine factors, resulting in hMSC differentiation at the gel-media interface. This zonal growth is reminiscent of in vivo bone growth mechanisms, where new bone tissue is produced in the outer layers of existing bone, such as at the periosteum,^49^ or within epiphyseal growth plates.^50^ These outcomes may be important for long term tissue regeneration, where newly formed bone tissue at the gel-media interface may be rapidly incorporated into the existing alveolar bone. If this were the case, then the presence of the alginate hydrogel may also act as a spacer, preventing invasion of endothelial cell layers into the defect whilst promoting bone regeneration. Nevertheless, these results highlight the importance of the dentine surface when modeling stem cell-laden injectable hydrogel therapies for oral healthcare.

To model the impact of microbiome on oral healthcare therapies, the effect of a representative oral bacteria *F. periodonticum* on hMSCs cultured in the 3D bioprinted construct was studied. The viability of *F. periodonticum* in 3D monoculture over 7 days suggest that obligate anaerobes may survive culture under aerobic conditions in hydrogels. This viability profile was attributed to environmental selection pressures, where a subpopulation was able to survive the new environment and thrive after 7 days. Alternatively, a reduction in oxygen diffusion across the hydrogel compared to free media may have resulted in a more suitable local oxygen concentration.^51^ The maintained viability in the presence of hMSCs suggests that either the known antimicrobial effects of hMSCs^52–55^ are dampened in this environment, or that the seeding multiplicity of infection (MOI) of *F. periodonticum* was too great. However, the presence of oral bacteria had a significant effect on hMSCs viability. The *F. periodonticum* infection led to hMSC death and exhibition of widespread stress responses. In general, the survival and proliferation of donor cells for periodontal tissue engineering is a key indicator of efficacy. In this work we demonstrate how oral bacteria affects the survival of stem cells, but cell proliferation was limited in the bioprinted constructs and not studied in the presence of bacteria. As such, this bioprinting approach would need to be further developed using bioink optimization to promote stem cell proliferation in the absence of oral bacteria. If successful, this would provide a route to high throughput testing of antimicrobial therapies and for the assessment of the impact of oral bacteria on regenerative oral cellular therapies.

## Conclusion

In this work, a stem cell-laden hydrogel-dentine interface was fabricated *via* 3D bioprinting with high cell viabilities and homogenous cell dispersions over 7 days of culture. The hMSCs encapsulated within the hydrogel could interact with the dentine surface, and the dentine surface was found to be osteoinductive in the absence of supplemented growth factors. However, the response in ECM production and tissue maturation was predominantly observed at the gel-media interface, distal to the gel-dentine interface. These results highlight the importance of including tissues from the wider niche that is being modeled, as these may impact on experimental outcomes. Furthermore, we have demonstrated a new bioprinting methodology for the 3D coculture of an obligate anaerobe *F. periodonticum* and hMSCs, which presents a possible avenue for the high throughput testing of oral healthcare therapies under infection. Accordingly, such 3D in vitro dental models could be invaluable tools to probe these behaviors and to further explore and understand the effect of dentine surfaces on injectable hydrogel-based therapies.

## Experimental section/methods

### Cell culture

Human mesenchymal stem cells (hMSCs) were isolated from the proximal femur bone marrow of consenting patients undergoing total hip replacement surgery at Southmead hospital according to Bristol Southmead Hospital Research Ethics Committee guidelines (REC reference: 20/LO/0614). Cell culture work was undertaken using laminar flow hoods (SAFE 2020, ThermoScientific), with incubation at 37 °C under a 5% CO_2_ atmosphere (HERACELL 240i, ThermoScientific). “Basal media” was prepared from low glucose DMEM supplemented with fetal bovine serum (10 v/v.%, Sigma-Aldrich), GlutaMax (2 mol/L, Invitrogen), and penicillin/streptomycin (100 units per mL/100 μg/mL, Sigma Aldrich). hMSCs were expanded in an “expansion media” consisting of basal media further supplemented with basic fibroblast growth factor 2 (5 ng/mL, Peprotech). For growth factor-induced hMSC differentiation, an “osteoinductive media” was prepared from basal media further supplemented with ascorbic acid (200 μmol/L, Sigma-Aldrich), bone morphogenic protein 2 (25 ng/mL, Gibco), dexamethasone (10 nmol/L, Sigma-Aldrich), and β-glycerophosphate (10 mmol/L, Sigma). All media used for culture of bioprinted constructs was further supplemented with CaCl_2_ (5 mmol/L).

Ascorbic Acid stock solutions (80 mmol/L) were prepared by dissolution of ascorbic acid 2-phosphate (69.5 mg, A8960, Sigma-Aldrich) in deionized water (3 mL) and filter sterilization (0.22 μm, 16534, Sartorius). Basic fibroblast growth factor 2 (FGF-2) stock solutions (10 μg/mL) were prepared by dissolution of basic human FGF (50 μg, 100-18B, Peprotech) in tris-buffered deionized water (4 mmol/L, 5 mL, pH = 7.6, 0.22 μm filter sterilized). Bone morphogenic protein 2 (BMP-2) stock solutions (100 μg/mL) were prepared by dissolution of recombinant BMP-2 (50 μg, PHC7145, Gibco) in 0.5 mL of a filter-sterilized solution of bovine serum albumin (1 mg/mL) in hydrochloric acid (4 mmol/L). β-Glycerophosphate stock solutions (1 mol/L) were prepared by dissolution of β-glycerophosphate disodium salt hydrate (1.08 g, G9422, Sigma-Aldrich) in deionized water (5 mL) and filter sterilization (0.22 μm). Basal ITS media composed of high glucose DMEM supplemented with penicillin/streptomycin (100 units per mL/100 mg/mL, P0781, Sigma-Aldrich), GlutaMAX (2 mmol/L, 35050038, Life Technologies), sodium pyruvate (1 v/v.%, 11360, Gibco) and insulin-transferrin-selenium (1 v/v.%, 41400045, Gibco) was prepared. Dexamethasone stock solutions (100 μmol/L) were prepared by dissolution of dexamethasone (3.925 mg, D4902, Sigma-Aldrich) in ethanol (1 mL), addition to basal ITS media (30 μL in 2.97 mL) and filter sterilized (0.22 μm).

### Substrate preparation

Acellular thin dentine disks were provided either by GlaxoSmithKline or the Tooth Tissue Bank (HTA license 12200, NHS REC reference, 16/NI/0192, Project 80, Bristol Dental School) for use in this work. The dentine disks were first subject to exfoliation using ascending grades of sandpaper (P400-P1200) with deionized water drop cast over the area; followed by ultrasonication (750 W, 20 kHz, amplitude 50%, 1 min duration, 2 s pulse on, 1 s pulse off, Sonics Vibra-Cell) in citric acid solution (0.5 w/v.% in deionized water, CO759, Sigma-Aldrich) and deionized water. To anchor the dentine slices within the well for 3D bioprinting, 3D printed inserts (PLA, Supplementary Fig. S13) were fabricated. βTCP (9.5 mm diameter, 3D BioTek, TCP48) disks were exfoliated using ascending grades of sandpaper (P400-P1200) with deionized water drop cast over the area to ensure reproducibility across samples. The dentine disks, βTCP disks and inserts were sterilized by incubation in 70% ethanol (overnight), wicked dry using lab roll and further sterilized by UV-C radiation on both faces (30 min each) in a laminar flow hood immediately before printing.

### Bioink preparation

A Pluronic F-127 stock solution (40 w/v.% in PBS, P2442, Sigma-Aldrich) was prepared and sterilized by autoclaving (121 °C, 45 min). Sodium Alginate (6 w.t.%, W20150, Lot# MKCC4541, Sigma-Aldrich) was added to basal media (60 v/v.%) and was mixed by hand. The gel was further mixed using a dual-asymmetric centrifuge (DAC, 3 500 r/min, 5–10 min until fully mixed, DAC 150.1 FVZ, Speedmixer). The gel was then sterilized by UV irradiation (>1 h) in a laminar flow hood. Pluronic F-127 stock solution (13 w/v.% final) was added using a positive displacement pipette, followed by dual-asymmetric centrifugation (1 500 r/min, 10–20 s until mixed). For cellular printing, cells were resuspended (7.5% final volume) and added to the gel, followed by dual-asymmetric centrifugation (1 500 r/min, 10 s). For acellular bioink, basal media (7.5% final volume) was added to the gel, followed by mixing using the DAC (1 500 r/min, 10 s).

### Bioprinting

A BioX (Cellink) 3D bioprinter was used for all bioprinting experiments in a temperature controlled lab (17 °C). A sterilization program on the printer (UV-C exposure within the printing enclosure) was run both before and after the printing process to ensure sterility within the printer, and the laminar flow hood sterilized by UV-C irradiation (1 h) prior to use. To load the cell-laden or acellular bioink into pneumatic syringes for 3D bioprinting, the ink was first loaded into a 5 mL syringe (Tenmo, SS*05SE1) using a sterile spatula and extruded into a 3 mL pneumatic syringe (7012074, Nordson) via a female-female Luer connector (11891120, Fisher-Scientific). The pneumatic syringe was then equipped with a needle (22 G, 2484538, Onecall), placed within the printhead and connected to the pneumatic tubing. STL files were generated using Autodesk Inventor and sliced using the BioX on-board slicer (100% infill density, rectilinear style pattern, 20 mm/s, 0.41 mm nozzle diameter). All constructs were printed into either a 24 or 48 well plate (Corning, 10732552 or 10065370 respectively) which was heated by the print bed (37 °C). Straight after printing, the samples were crosslinked in DMEM supplemented with CaCl_2_ (100 mmol/L, 10 min, RT, 349615000, Acros Organics, filter sterilized). The samples were then cultured under standard conditions, with media supplemented with CaCl_2_ (5 mM, filter sterilized).

### Cell viability studies

Live/Dead staining was performed using a commercially available kit (L3224, ThermoFisher). Staining solution was prepared fresh by addition of Calcein AM (0.5 μL/mL) and ethidium homodimer-1 (2 μL/mL) to pre-warmed PBS and wrapped in foil to prevent photobleaching. Bioprinted constructs cultured in expansion media for 1 or 7 days were washed with PBS (pre-warmed to 37 °C) and incubated in staining solution (0.5 mL, 30 min, 37 °C, wrapped in foil). After incubation, the samples were imaged using a widefield microscope (Calcein AM filter = GFP, ethidium homodimer-1 filter = Texas Red, Leica DMI6000 inverted epifluorescence microscope, equipped with a Leica LASX live cell imaging workstation and a Photometrics Prime 95B sCMOS camera). Cell counting was performed on widefield image stacks using FIJI.

### Cell distribution studies

3D iterative thresholding of the live channel from widefield stacks used for cell viability studies was performed using the 3D manager package for FIJI (3D iterative thresholding plugin: min_vol_pix = 100, max_vol_pix = 20,000, min_threshold = 10, min_contrast = 0, criteria_method = MSER, threshold_method = step, segment_results = All, value_method = 10).^56^ Image sets were frequently recorded with a volume above and below the gel in z to ensure that the maximal volume of gel was imaged. This often led to void regions in the z axis where no cells were present. These regions were first determined manually on the original image stacks, and the corresponding distances were removed from the beginning and end of the z data. Histograms along each axis were then plotted with the calculated xyz coordinates to yield live cell distributions (distance normalized to dimensions of imaged volume). Distributions are presented as the average of all cell positions taken over at least 34 stacks.

### Immunofluorescence microscopy

A “wash buffer” (WB, 0.05 v/v.% Tween 20 in PBS, T2700, Sigma-Aldrich), a “permeabilizing buffer” (PB, 0.1 v/v.% Triton X-100 in PBS, T8787, Sigma-Aldrich) and a “blocking buffer” (BB, 1 w/v.% bovine serum albumin in PBS, A7638, Sigma-Aldrich) were prepared fresh and filter sterilized (0.22 μm). DAPI staining solution was prepared from a DAPI stock solution (0.1 v/v.% in PBS, Millipore, 90229) and was wrapped in foil. For actin staining, a dual-staining solution consisting of anti-phalloidin TRITC solution (4 v/v.% of a 60 μg/mL stock, 90228, Millipore) and DAPI staining solution in PBS was prepared fresh just before use. Samples were washed with WB (500 μL, 3 times, 10 min each) and permeabilized with PB (500 μL, 10 min). Samples were washed with WB (500 μL, 3 times, 10 min each) and incubated (overnight, 4 °C) in dual-staining solution. The samples were then washed with WB (500 μL, 3 times, 10 min each), transferred to a confocal dish flooded with PBS and imaged using a Leica SP8 AOBS confocal laser scanning microscope attached to a Leica DMi8 inverted epifluorescence microscope.

For collagen staining, anti-collagen I primary antibody solution (0.05 v/v.% in BB, Invitrogen, MA1-26771), mouse IgG solution (0.3 v/v.% in BB, BioVision, 1265-100), FITC GoatxMouse IgG secondary antibody solution (0.4 v/v.% in PBS, Millipore, AP124F), and DAPI staining solution were prepared fresh before use. Samples were washed with WB (500 μL, 3 times, 10 min each) before incubation in BB (500 μL, 30 min). Samples were incubated in primary antibody solution (500 μL, 4 °C, overnight, wrapped in foil). Isotype controls were prepared by incubation in mouse IgG solution (500 μL, 4 °C, overnight) instead of primary antibody solution. No primary antibody controls were prepared by incubation in blocking buffer (500 μL, 4 °C, overnight) instead of primary antibody solution. The next day, samples were washed with WB (500 μL, 3 times, 10–15 min each) before incubation (45 min, RT, wrapped in foil) in secondary antibody solution (500 μL). No secondary antibody controls were prepared by incubation in PBS instead of secondary antibody solution. The samples were then washed with WB (500 μL, 3 times, 10–15 min) before incubation in DAPI staining solution (500 μL, 30 min, RT, wrapped in foil). The samples were then washed with WB (500 μL, 3 times, 10–15 min each). The samples were then placed in confocal dishes and submerged with PBS for imaging using a confocal microscope (Leica SP8 AOBS confocal laser scanning microscope attached to a Leica DM I6000 inverted epifluorescence microscope).

### Gene expression analysis

Cetyltrimethyl ammonium bromide buffer solution (CTAB, Promega, MC1411) was freshly supplemented with 1 v/v.% β-mercaptoethanol and pre-warmed to 65 °C. Bioprinted constructs were washed with PBS and pooled into an RNase-free Eppendorf (AM12400, Fisher). The samples were then dissolved using EDTA (as described in section 2.9.2.2.1) to yield a cell pellet prior to addition of CTAB buffer (600 μL). The samples were then homogenized by passing through a 21 G needle (>20 passes, ThermoFisher, 10472204) and incubated (65 °C, 15 min) with frequent mixing by inversion. Debris was isolated by centrifugation (14 000 × *g*, 2 min, RT), and the supernatant transferred to a new Eppendorf. Chloroform extraction was performed by addition of chloroform (100 v/v.%, VWR, 22705.323) to the supernatant and mixed by inversion. The mixture was then centrifuged (14 000 × *g*, 2 min, RT) and the supernatant was carefully 62 aspirated without disturbing the interfacial region. Chloroform extraction was repeated one further time, resulting in a clear interfacial region.

Nucleic acid precipitation was performed by addition of either ethanol (2–2.5× volume) and sodium acetate (3 mol/L, 0.1× total volume, 15480217, Alfa Aesar) to the supernatant, mixed by inversion, and incubation (−80 °C, overnight). The sample was thawed on ice, and the white precipitate was isolated by centrifugation (14 000 × *g*, 30 min, 4 °C).

The precipitate was then washed with ethanol (70 v/v.% in RNase-free water, ice cold), re-isolated by centrifugation (14,000 × *g*, 15 min, 4 °C), and the supernatant was removed. This wash step was repeated two further times. The samples were then incubated (5–10 min, RT) to allow residual ethanol to evaporate. Samples were then dissolved in RNase-free water (750024, Fisher) were further purified using RNeasy Mini kit (Qiagen, 74104) following manufacturer’s instructions. Samples were eluted with DEPC-treated deionized water (46-2224, Intvitrogen). cDNA was synthesized using a High-Capacity cDNA Reverse Transcription Kit (ThermoFisher) according to manufacturer’s instructions using a SensoQuest Labcycler (Geneflow). qPCR was performed using a Step One Plus RT- PCR system (Applied Biosystems) and the following TaqMan probes: ALPL (Hs01029144_m1), BGLAP (Hs01587814_g1), COL1A2 (Hs01028969_m1), ELF1 (Hs01111177_m1), RUNX2 (Hs01047973_m1), YWHAZ (Hs01122445_g1).

Samples were analyzed using the ΔΔCT method, with the geometric mean of ELF1 and YWHAZ used as the reference. Statistical analysis for autoinduction was performed on ΔCT values using paired t-tests, whilst statistical analysis for comparison between groups was performed on ΔΔCT values using a 2-way ANOVA.

### Histological analysis

Samples were first fixed in PFA solution (4 w/v.% paraformaldehyde in PBS, 2 h, RT). The samples were then immersed in ascending grades of ethanol (30, 50, 70, 90, 100 v/v.% in deionized water, 20 min each) before incubation in London Resin white (LRW), 50 v/v.% in ethanol, overnight, L9774, Sigma-Aldrich). The samples were incubated in three further changes of LR white (100%, 2 h each) before transfer into a TAAB capsule. The capsule was sealed and incubated (60 °C, ~2 days) to cure. If the sample required reorientation to section along a desired axis, the block was cut parallel to the desired axis using a hacksaw and remounted onto the same block base using commercial superglue. The block was then sectioned using an ultramicrotome (1 μm, Leica EM UC6), floated onto glass slides using deionized water and placed on a heating block (~80 °C) until fully dry.

For calcium staining, sections were incubated (2.5 min, RT) with alizarin red solution (2 w/v.% in deionized water, pH = 4.25, filtered) dropcast to cover the section. Excess stain was then removed by careful blotting. For glycosaminoglycan staining, sections were incubated (20 min, RT) in acetic acid solution (1 v/v.% in deionized water, pH = 2.20). Sections were then incubated (10 min, RT) in safranin-O solution (0.1 w/v.% in deionized water, pH = 2.20), with excess stain removed by washing with deionized water. Sections were allowed to dry and imaged using a widefield microscope (Leica DMI6000 inverted epifluorescence microscope, equipped with a Leica LASX live cell imaging workstation and a Leica DFC420C color camera).

### Coculture studies

For bacterial coculture studies, all cell culture was performed without penicillin/streptomycin. *Fusobacterium periodonticum* (ATCC33693, strain 2B3) were inoculated and grown (2 days, 37 C) on fastidious anaerobic agar plates under anaerobic conditions (Oxoid Anaerogen, ThermoScientific) in a sealed container. *F. periodonticum* were then suspended in PBS (pre-warmed), and the concentration determined by absorbance (OD 600 = 1 corresponding to 10^8^ cells per mL, GENESYS 6, ThermoSpectronic). For inoculation in the bioink, a small volume of basal media was first omitted from the alginate-gel formation step prior to UV sterilization. *F. periodonticum* (3 × 10^7^ cells per mL, MOI = 10) was suspended in an equivalent volume and added to the bioink either alongside hMSCs or the last volume of basal media for acellular ink. The bioink was then mixed by dual asymmetric centrifugation (1 500 r/min, 15 s), and bioprinted using an INKREDIBLE+ (Cellink). All samples were subsequently cultured under aerobic conditions.

Samples inoculated with *F. periodonticum* were stained using the BacLight Bacterial Viability kit (L7007, ThermoFisher) according to manufacturer’s instructions, and imaged using a confocal microscope (Leica SP8 AOBS confocal laser scanning microscope attached to a Leica DM I6000 inverted epifluorescence microscope). Semi-quantitative viability analysis of *F. periodonticum* was performed on image stacks via thresholding and pixel counting. Viability analysis of hMSCs was performed using the analyze particles plugin in FIJI.

### Supplementary information

Supplementary information

## Data Availability

All data used in this article is available in the University of Bristol’s Research Data Storage Facility and will be made available upon the publication of the article.
