# Supplementary material for: A 3D In-vitro model of the human dentine interface shows long-range osteoinduction from the dentine surface
Source: Int J Oral Sci. 2024 May 11;16:37. doi: 10.1038/s41368-024-00298-9 (PMC11088668; doi:10.1038/s41368-024-00298-9)
Supplement: Supplementary file 1 — Supplementary information [file 41368_2024_298_MOESM1_ESM.docx]

Supporting Information

A 3D In-vitro Model of the Dentine Interface Shows Long-Range Osteoinduction from the Dentine Surface.

William Macalester, Asme Boussahel*, Rafael Moreno, Mark R Shannon, Nicola West, Darryl Hill, Adam Perriman*

**Supplementary methods**

***Analysis of macromolecular diffusion through alginate and the bioink:*** The determination of the effective diffusion coefficient (D_eff_) of tetramethylrhodamine-coupled bovine serum albumin (BSA-TAMRA) in both alginate and the bioink was performed according to the protocol described by Huesbch et al.

To synthesize BSA-TAMRA, NHS-rhodamine (159 μL of 10 mg mL^-1^ stock in DMSO, 46406, ThermoFisher) was conjugated to BSA (50 mg, ~66 kDa, A7638, Sigma-Aldrich) by reaction (4 °C, 22 hours, wrapped in foil) in bicarbonate buffer (5 mL, 0.1 M NaHCO3 in deionized water, S6014, Sigma-Aldrich, pH = 8.5). To remove unreacted components, the products were purified using a Sephadex® G-25 desalting column. The degree of labelling was determined by UV-vis spectrophotometry.

For BSA-TAMRA diffusion studies, acellular bioink and alginate samples were prepared without GlutaMax and FBS added to basal media. After Pluronic F127 or DMEM respectively was spun into the gel, BSA-TAMRA was added to yield a final concentration of 1 mg / mL, and the samples kept wrapped in foil henceforth to prevent photobleaching of the fluorophore. The crosslinking solution and expansion media were supplemented with BSA-TAMRA (1 mg / mL), and all samples were taken after incubation (overnight, 37 °C) in 5 mM expansion media.

Samples were measured using digital calipers and transferred to a 6 well plate (1 sample per well). The samples were then incubated in low glucose DMEM (phenol free, + 5 mM CaCl_2_, + P/S, 9-10 mL) on an orbital shaker (30 rpm, 37 °C, 5 % CO_2_). The lowest shaking speed was used to remove complications that may arise due to boundary-layer effects whilst minimizing effect on bulk diffusion. Aliquots (100 μL) were taken from the center of the wells over 4 hours, and the endpoint fluorescence recorded (Ex/Em 552/575 nm, BioTek Synergy Neo2 Hybrid Multi-Mode Reader). The readings were then compared to a standard curve of known BSA-TAMRA concentrations. The D_eff_ was then calculated using equation (1).

$D_{eff}=\pi\left( \frac{m*V_{media bath}}{2 c_{matrix}A_{matrix}} \right)^{2}$ (1)

The semi-infinite slab approximation was validated over the range of timepoints used by least-squares fitting of the linear regression (all R^2^ > 0.90). Statistical analysis was performed using a Welch’s t-test (n=5). For comparison, Pluen et al. found the D_eff_ of BSA in water to be 6.4 ± 0.4 x10^-11^ m^2^ s^-1^.

***Unconfined Compression Analysis****:* Bioprinted constructs were crosslinked by incubation in 100 mM CaCl_2_ overnight, followed by incubation overnight with expansion media (+ 5 mM CaCl_2_) under tissue culture conditions before compression analysis. All mechanical testing was performed using a STARRET FMS-500-L2 Force Measurement System (The L.S. Starrett Company Ltd, UK) fitted with a 10 N load cell at room temperature. Unconfined compression testing was performed at a rate of 1 mm min^-1^. The Young’s modulus was calculated by linear regression analysis of the elastic region.

***FTIR analysis of bioprinted constructs****:* Cell laden constructs were bioprinted onto tissue culture plastic and cultured for 28 days with either expansion or osteoinductive media. 4 constructs were pooled per sample, washed with PBS, and lyophilized. Samples were then incubated with EDTA (50 mM, in PBS) until the alginate network was disassembled. The insoluble fraction was isolated by centrifugation (13000 g, 3 mins, RT) and washed with PBS. The insoluble fraction was then isolated by centrifugation (13000 g, 3 mins, RT) and allowed to dry. FTIR spectra of the samples were taken using a spectrum one FTIR spectrometer (Perkin Elmer) equipped with an attenuated total reflectance accessory between the range of 500 - 4000 cm^-1^ using transmission mode. Spectra were recorded with 50 scans accumulation at 0.2 cm s^-1^ and 0.5 cm^-1^ resolution. A blank background was recorded and subtracted from the measured spectra. IR: *ν* = 3278 (m), 3072 (m), 2930 (s), 1634 (m), 1548 (m), 1445 (w), 1410 (m), 1260 (s), 1012 (s), 961 (m), 871 (s), 600 (s), 557 cm^−1^ (s).^[52]^

***Powder XRD analysis of bioprinted constructs****:* Cell laden bioprinted samples were cultured for 28 days in either expansion or osteoinductive media. Samples were washed with PBS, pooled (8 samples per condition) and lyophilized. The sample was then ground into a powder, deposited onto a film of silicon grease that was prepared on a silicon wafer and tapped clean with a clean glass slide. The sample was then submitted to the X-ray crystallography facility for powder X-ray diffraction using a D8 powder diffractometer (Bruker), using Cu Kα radiation with a scan range 2θ of 5 to 80 ° at a resolution of 0.5 ° and residence time of 0.2s. For analysis, background was subtracted using MATCH! XRD analysis software.

***Scanning electron microscopy with energy dispersive X-ray analysis:*** Cell laden bioprinted samples were cultured for 28 days in osteoinductive media prior to fixation and embedding in LR White. Sections (1 μm) along the xy plane at the gel-media interface were floated onto a glass coverslip. The coverslip was then mounted and grounded on a chuck using conductive tape. The sample was then sputter coated with silver (~5 nm) and imaged using a JSM-IT300 (JEOL), equipped with an X-Max 80 energy dispersive spectrometer (Oxford Instruments). Samples were imaged using either a secondary electron or backscatter detector.

***Microcomputed Tomography:*** Microcomputed tomography (µCT) measurements were performed on PE, PO, DE and DO samples after 28 days culture and embedding in LRW. All measurements were performed using a CT scanner (XTH225ST, Nikon) with a 3 micron focal spot size at 35 kV by Katie Smith at the Bristol Composites Institute. Projections were recorded across 360° rotation and reconstructed using VG Studio MAX 3.3.

***Characterization of bacteria after culture in the bioink****:* For Gram staining, four constructs were inoculated with *F. periodonticum* (MOI = 10) via dual asymmetric centrifugation and cultured in expansion media for 7 days. The constructs were pooled into an Eppendorf, and PBS (300 μL, pre-warmed) was added. An *F. periodonticum* suspension was prepared by mechanical fragmentation of the constructs using RNase/DNase free pestles (ARG1532, SLS). The suspension was transferred onto a glass slide using either a loop, or a pipette (30 μL) followed by spreading with a loop and allowed to air dry. The sample was then heat fixed and allowed to cool prior to staining. The sample was incubated with crystal violet solution (PL.7001, Pro-Lab Diagnostics) for 1 minute and the excess tipped away. The sample was then incubated with Gram’s iodine solution (PL.7004, Pro-Lab Diagnostics) for 1 minute, and the slide washed with deionized water. The sample was briefly incubated in Gram’s differentiator (PL.7007, Pro-Lab Diagnostics) to remove excess stain, and then washed with deionized water. The sample was counterstained with Safranin-O solution (PL.7013, Pro-Lab Diagnostics), briefly washed with deionized water and left to dry. Images were recorded using a phone camera using a 100x objective.

**Supplementary figures**


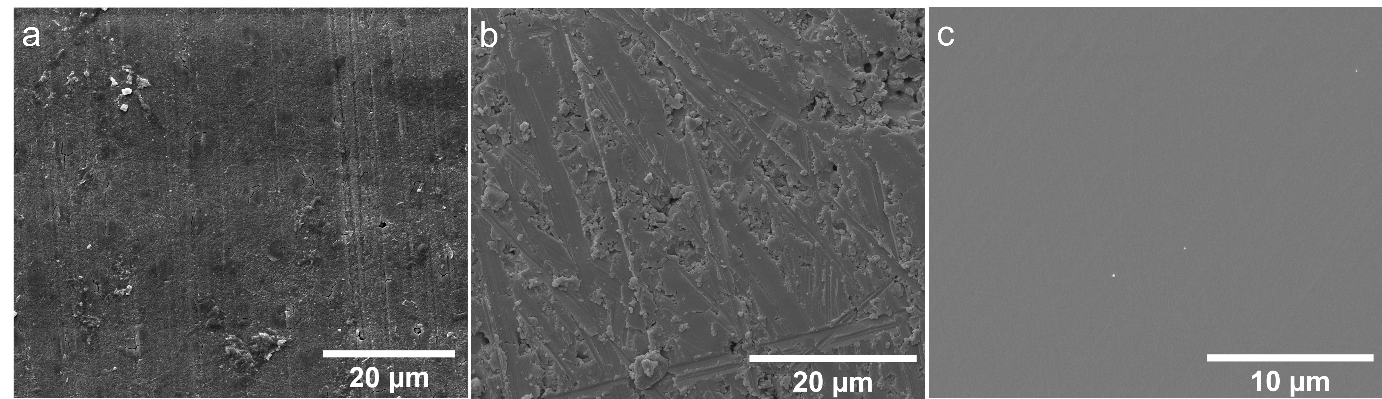


**Figure S1.** SEM micrographs of untreated dentine (a), βTCP (b) and tissue culture plastic (c). A smear layer present on the dentine surface occludes the tubules (dark shadows).


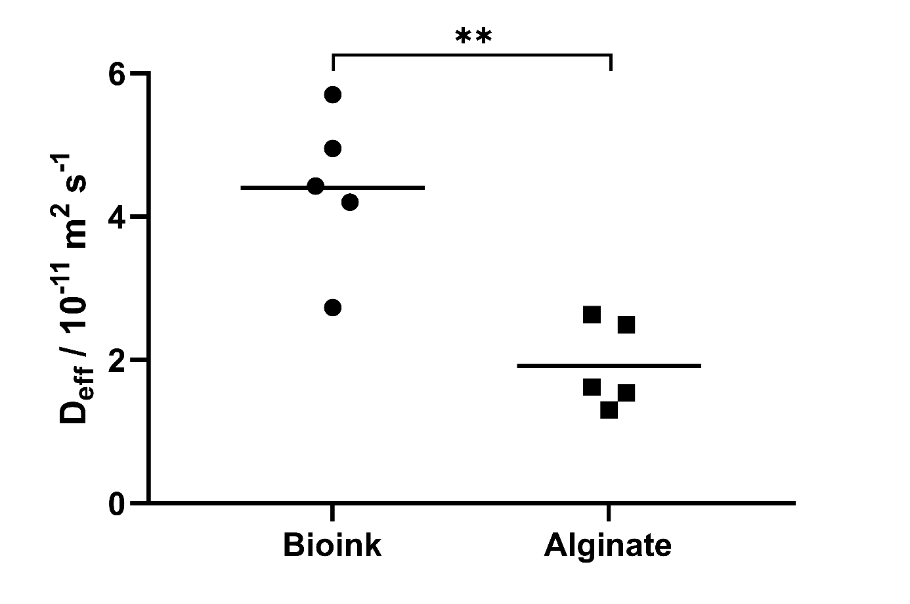


**Figure S2.** The Deff of BSA-TAMRA in either the bioink or alginate hydrogel, as calculated using the semi-infinite slab approximation. The Deff of BSA-TAMRA was significantly greater in bioink than alginate, suggesting that the templating of a microporous network results in greater mass transport through the gel. Statistical significance was determined by Welch’s t-test (** = p < 0.01, n = 5). Bar represents the mean.


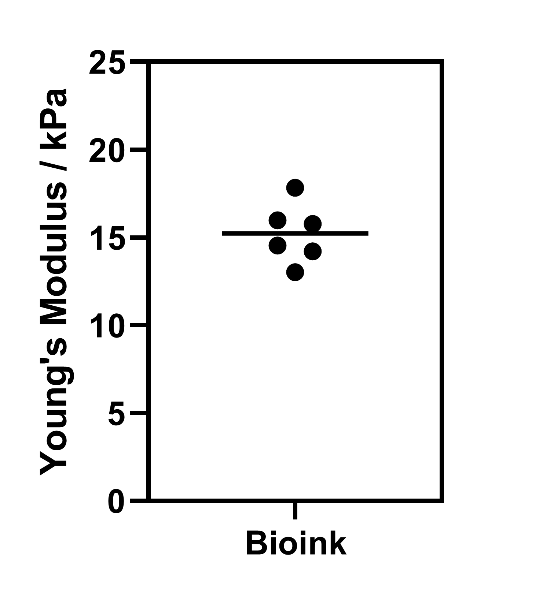


**Figure S3.** Young’s moduli of the bioink under tissue culture conditions. Bar represents the mean.


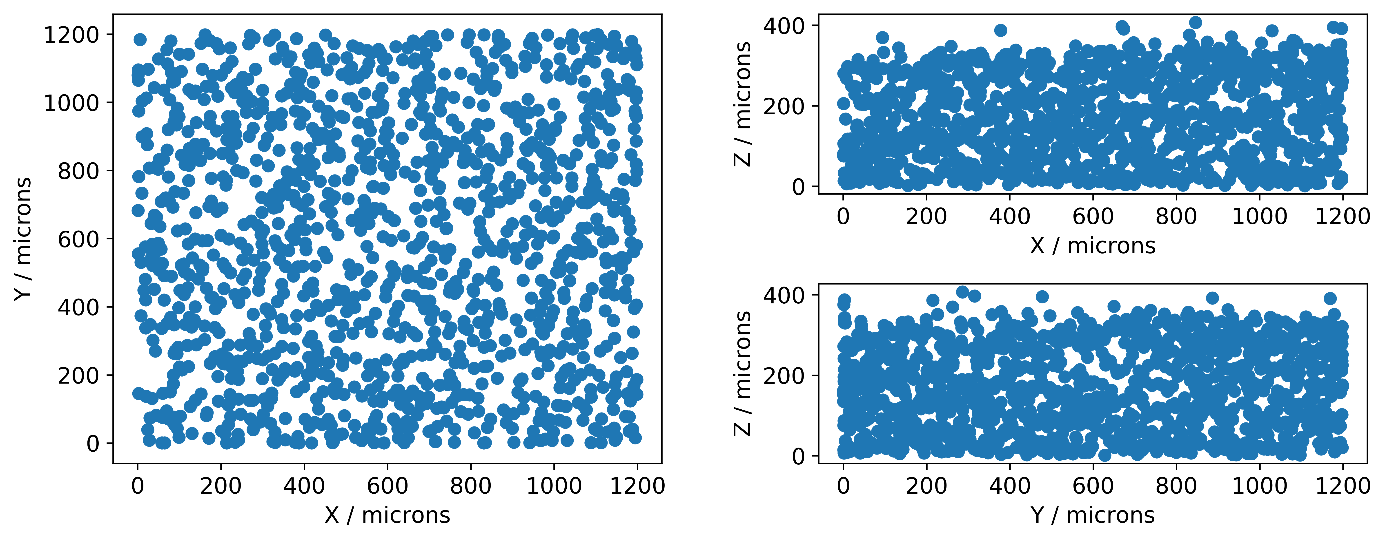


**Figure S4.** Representative plots of individual cell distribution across xy, xz and yz faces, taken from a sample bioprinted onto tissue culture plastic and cultured with expansion media for 1 day. Cells were well distributed across the imaged volume.


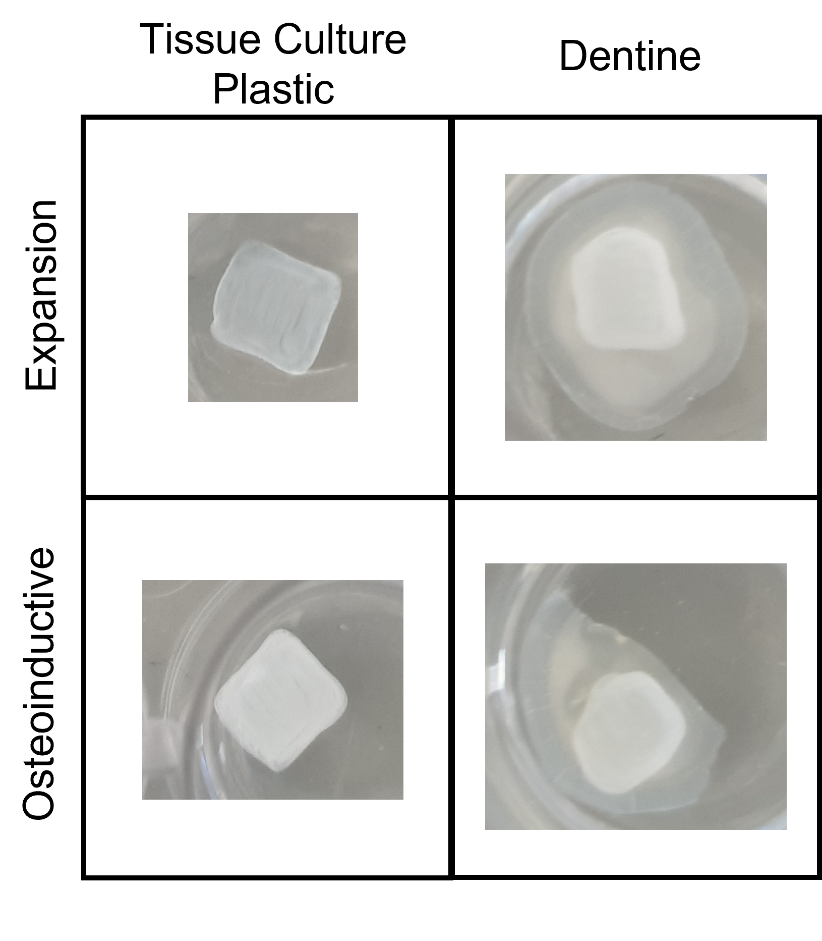


**Figure S5.** Pictures of samples after 4 weeks culture. There is an increase in opacity for samples cultured with expansion media on dentine compared to tissue culture plastic that was also observed when removed from the dentine slice.


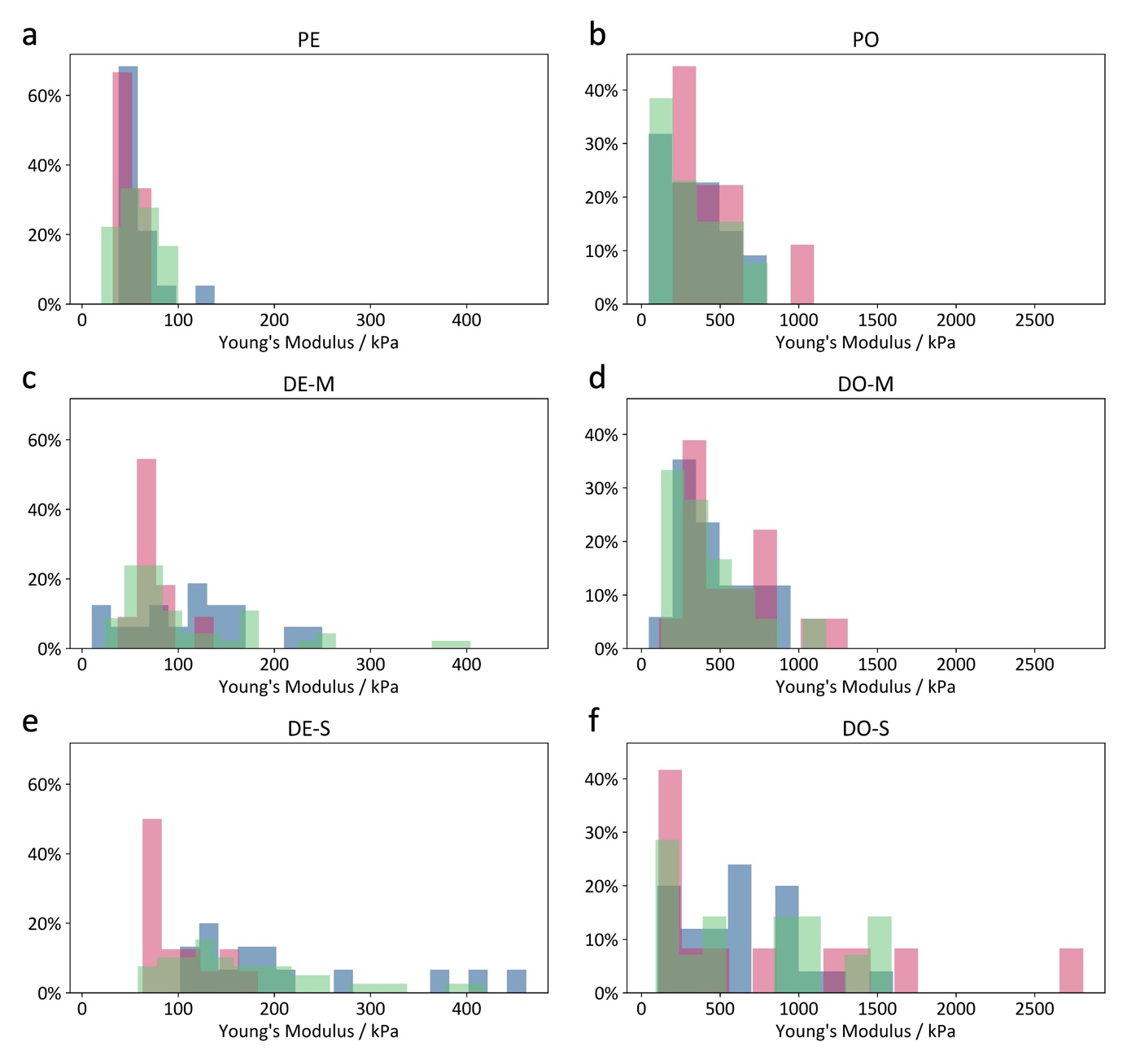


**Figure S6.** Nanoindentation analysis of bioprinted constructs after 5 weeks of culture on either tissue culture plastic (a, b) or dentine (c, d, e, f) in either expansion (a, c, e, bin width = 20 kPa) or osteoinductive (b, d, f, bin width = 150 kPa) media. For samples cultured on dentine, nanoindentation analysis was performed at either the gel-media interface (c, d) or the gel-dentine interface (e, f). Different colours represent different biological repeats (N=

β-TCP


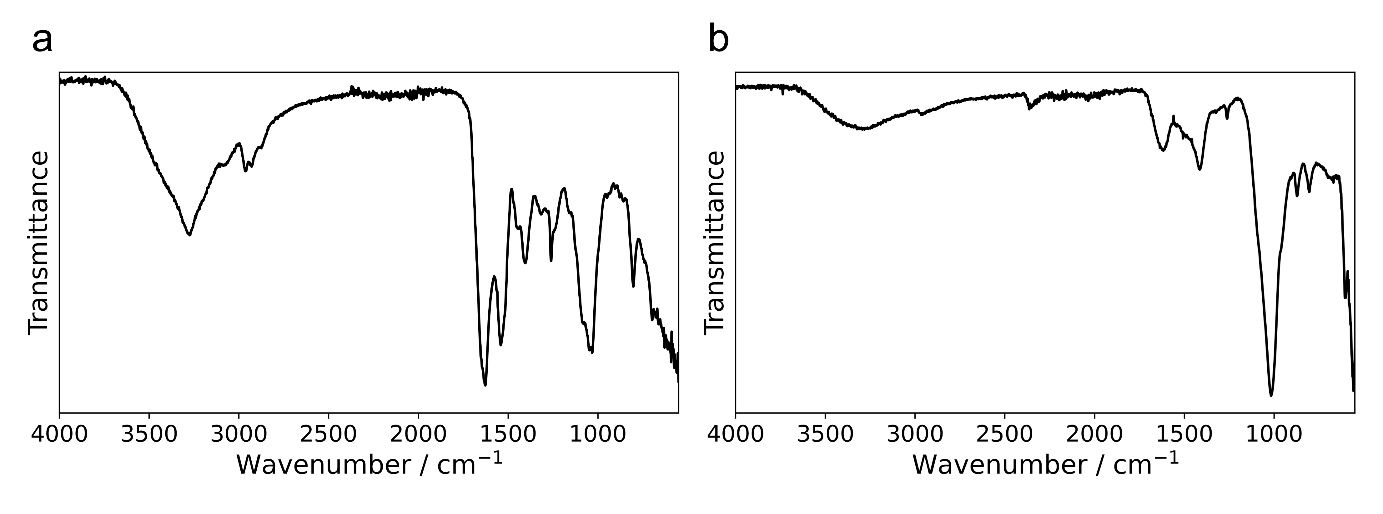


**Figure S 7..** Representative FTIR spectra of cell laden constructs bioprinted onto culture plastic and cultured for 28 days in either expansion (a) or osteoinductive media (b). Characteristic peaks for hydroxyapatite (557, 600, 871, 961, 1012 cm-1) in bone were all observed for the osteoinductive samples, but were absent for samples cultured in expansion media.


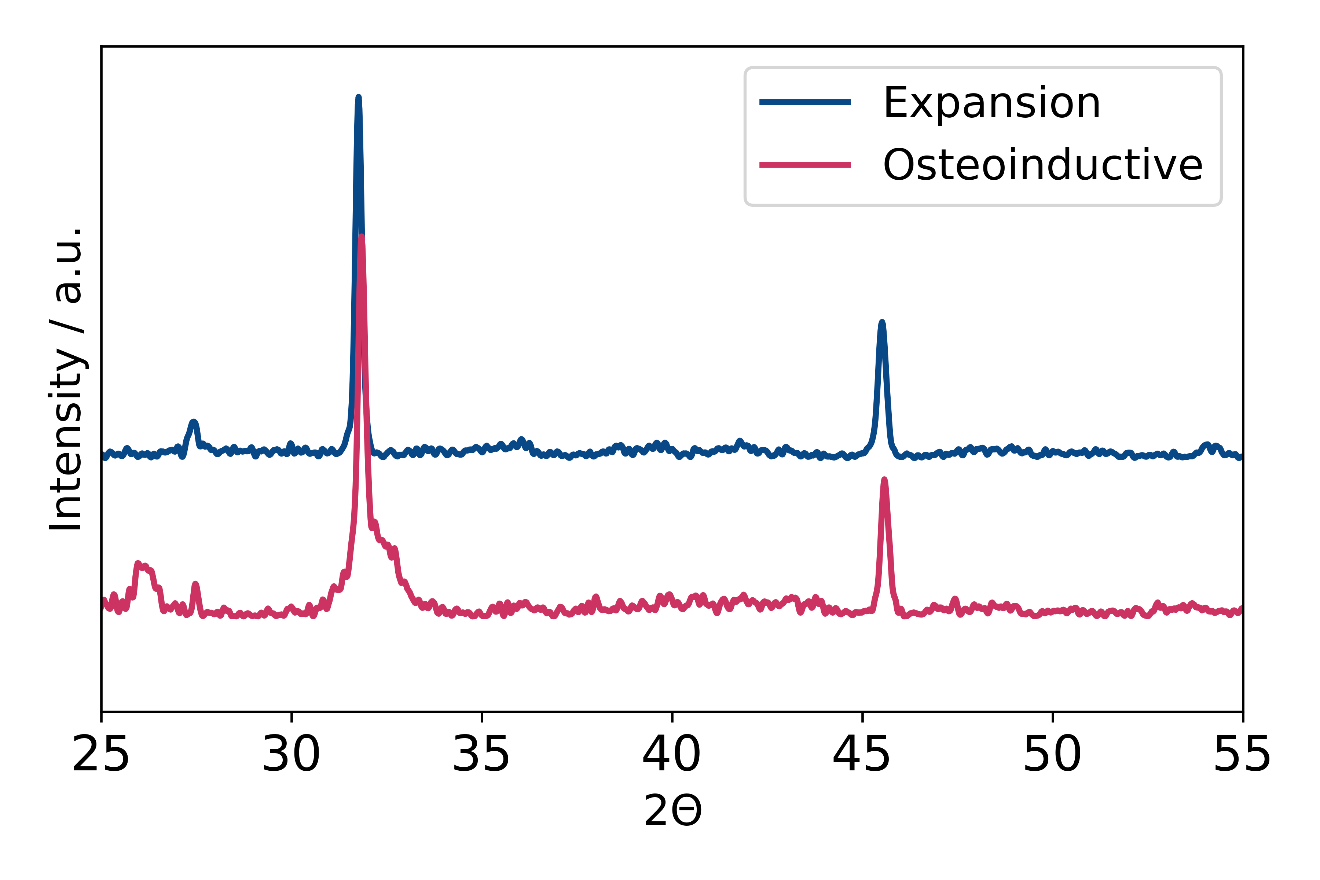


**Figure S 8..** Powder X-ray diffraction analysis of cell laded bioprinted samples after culture in either expansion (blue) or osteoinductive (red) media for 28 days. Sharp peaks at 31.8° and 45.5° 2θ correspond to residual NaCl from PBS wash steps. Broad peaks corresponding to hydroxyapatite (26°, 32° 2θ) were only observed for sample in osteoinductive media.


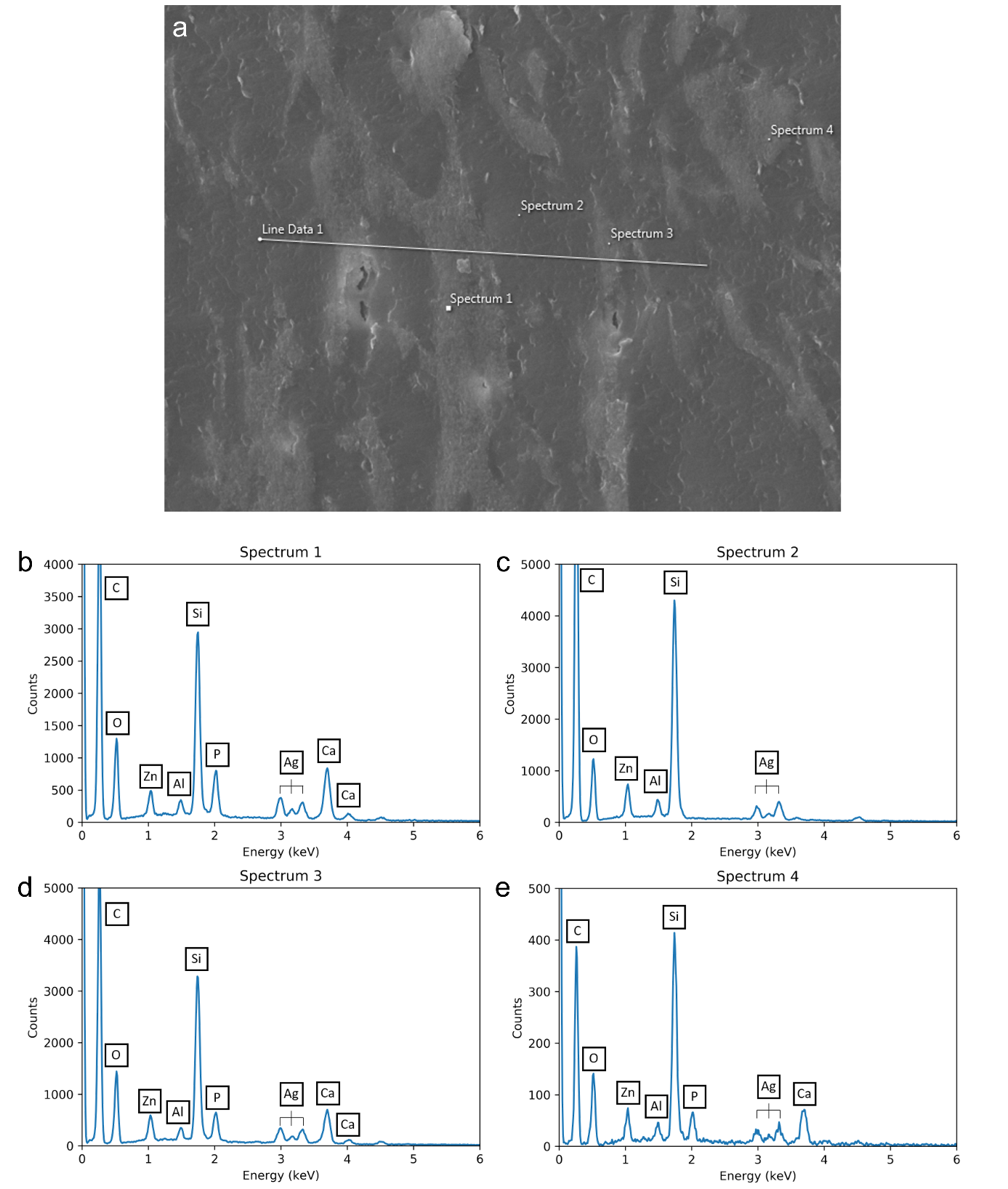


**Figure S 9..** SEM and EDX analysis of a cell laden sample bioprinted onto tissue culture plastic and cultured for 28 days in osteoinductive media. The sample was embedded in LRW, sectioned (1 μm) and coated with silver. a) Secondary electron detector with overlay of regions used for EDX spot spectra, corresponding to either high (b, d, e) or low intensity (c) regions. Calcium and phosphorus peaks were observed in high density regions, and absent in the low density region.


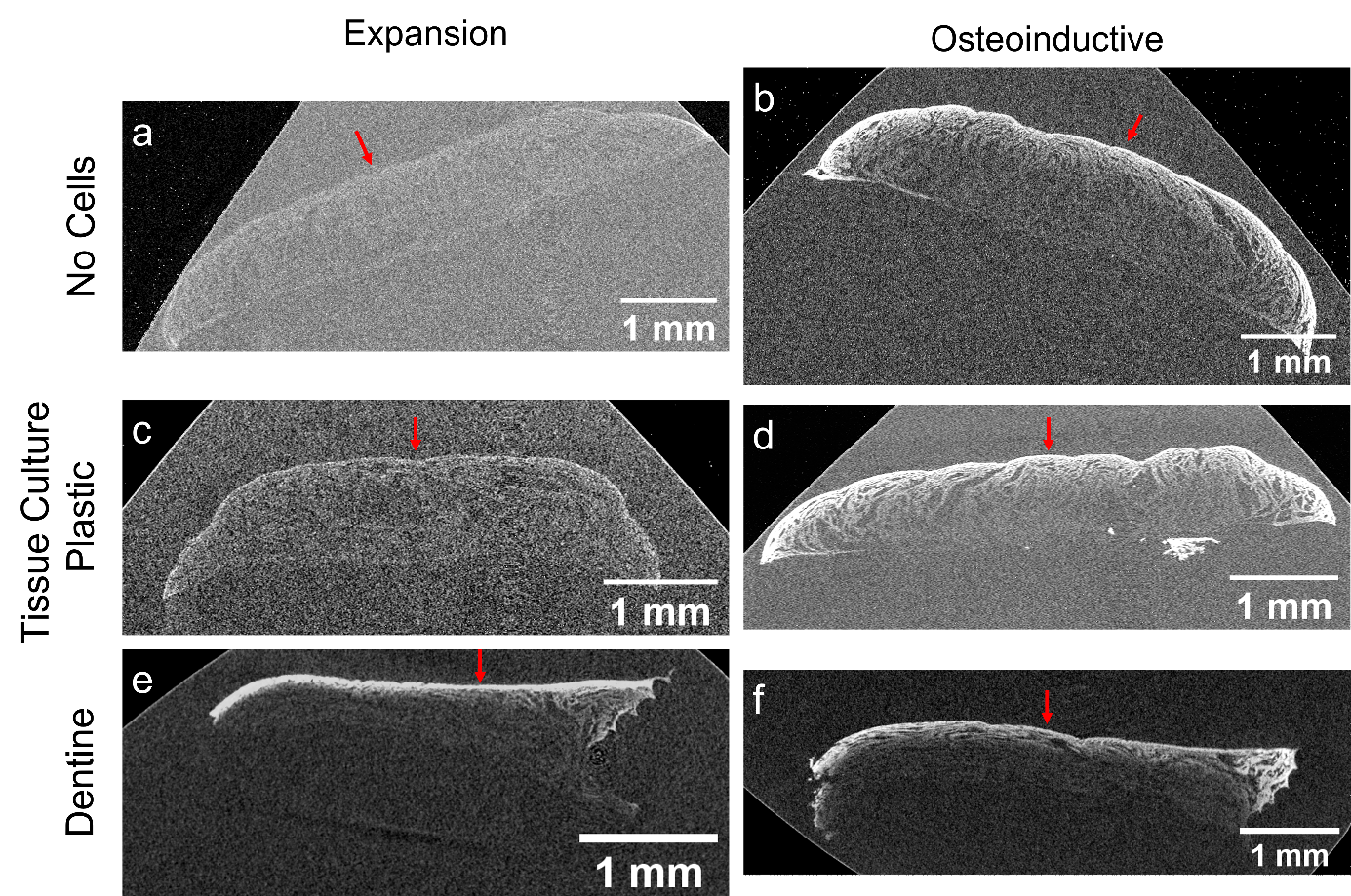


**Figure S 10.** Representative μCT images of constructs cultured for 28 days in either expansion (a, c, e) or osteoinductive media (b, d, f) and embedded in London Resin White. Acellular controls in tissue culture plastic revealed minimal contrast to resin for expansion media (a), whereas there was contrast at the gel-media interface for osteoinductive (b). Samples bioprinted onto tissue culture plastic yielded minimal contrast in expansion media (c) and regions of high contrast at the gel-media interface for osteoinductive (d). Samples bioprinted onto dentine both yielded regions of high contrast at the gel-media interface, but minimal contrast at the gel-dentine interface. Red arrows indicate the gel-media interface. N = 1


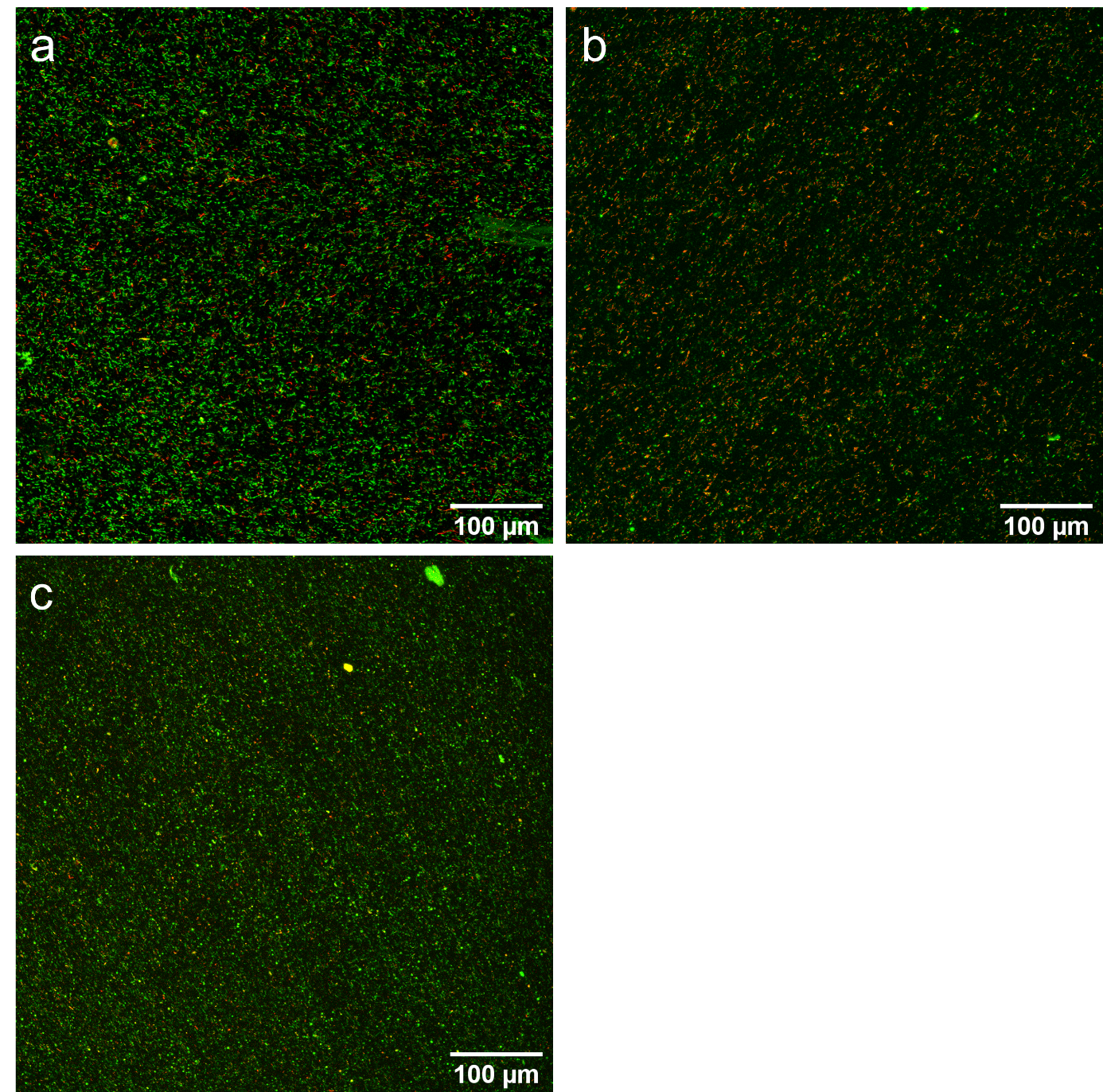


**Figure S 11.** Confocal micrographs of total nucleic acid (green, Syto9) / dead (red, propidium iodide) staining of F. periodonticum after culture for 0 (a), 1 (b) or 7 (c) days. Bioprinted constructs were inoculated with F. periodonticum with an MOI of 10 by centrifugation using the DAC. Small clusters of staining were observed homogenously throughout the bioprinted constructs, with more dead cells observed at day 1 (b) than the other two timepoints.


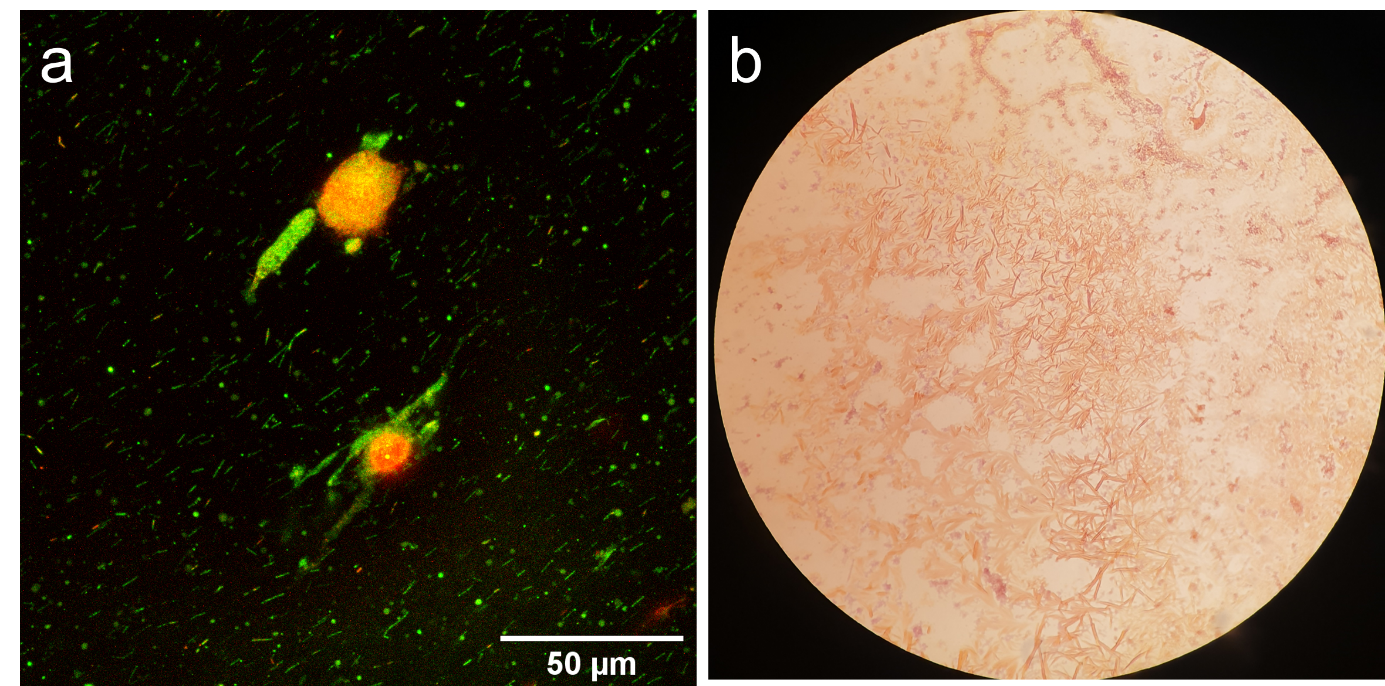


**Figure S12.** Characterization of F. periodonticum after 7 days coculture within the bioprinted constructs (MOI = 10). a) Confocal micrographs of live (green, Syto9) / dead (red, propidium iodide) staining revealed rod-like morphologies within the bioprinted constructs. b) Gram staining of the bioprinted construct yielded gram-negative rods. Image was taken through a 100x objective.


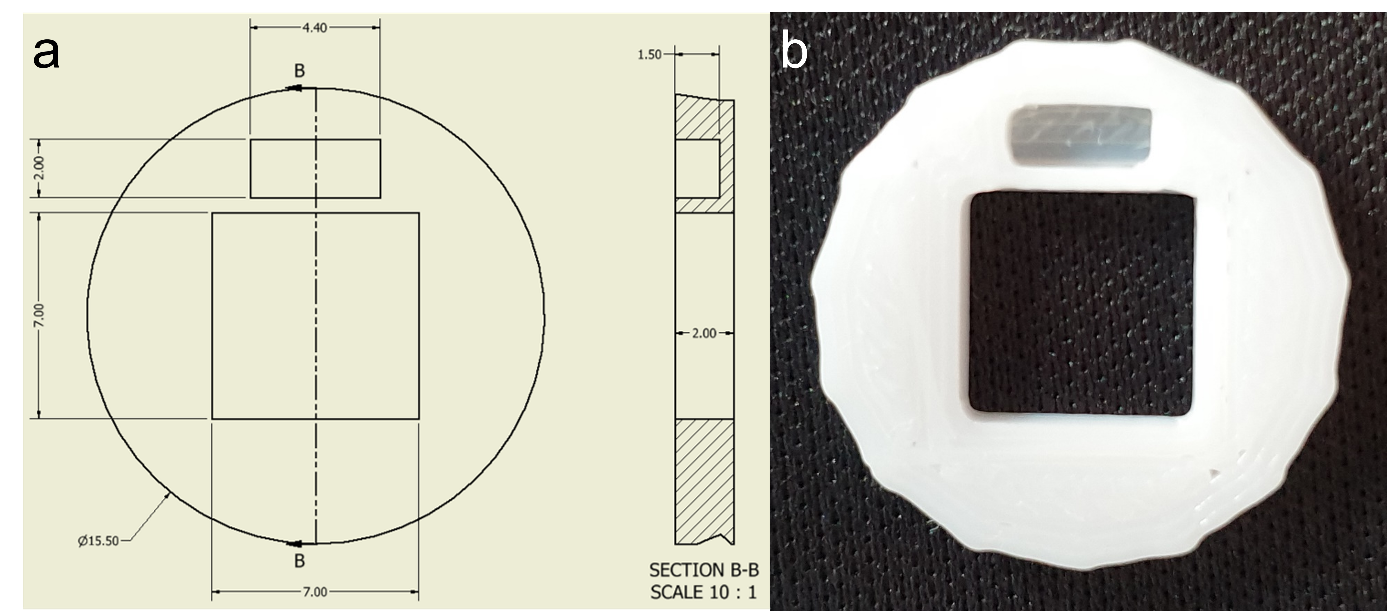


**Figure S13.** The insert used for anchoring dentine slices in the well plate during bioprinting. a) Schematic of the insert. A window in the center of the insert allows for the syringe to pass through and print directly onto the dentine surface. A small ridge was incorporated to aid in placement and removal of the insert. b) A picture of the insert, 3D printed with PLA.
